# Phenylalanine Ammonia-Lyase: A Key Gene for Color Discrimination of Edible Mushroom *Flammulina velutipes*

**DOI:** 10.3390/jof9030339

**Published:** 2023-03-09

**Authors:** Ji-Hoon Im, Hye-Won Yu, Che-Hwon Park, Jin-Woo Kim, Ju-Hyeon Shin, Kab-Yeul Jang, Young-Jin Park

**Affiliations:** 1 Mushroom Research Division, National Institute of Horticultural and Herbal Science, Rural Development Administration, 92, Bisan-ro, Eumseong-gun 27709, Republic of Korea; 2 Department of Medicinal Biosciences, Research Institute for Biomedical & Health Science, College of Biomedical and Health Science, Konkuk University, 268 Chungwon-daero, Chungju-si 27478, Republic of Korea

**Keywords:** *Flammulina velutipes*, fruiting body color, genome, phenylalanine ammonia-lyase

## Abstract

In nature; *Flammulina velutipes*, also known as winter mushrooms, vary in the color of their fruiting bodies, from black, yellow, pale yellow, or beige to white. The purpose of this study was to compare the genome sequences of different colored strains of *F. velutipes* and to identify variations in the genes associated with fruiting body color. Comparative genomics of six *F. velutipes* strains revealed 70 white-strain-specific variations, including single nucleotide polymorphisms (SNPs) and insertions/deletions (indels), in the genome sequences. Among them, 36 variations were located in the open reading frames, and only one variation was identified as a mutation with a disruptive in-frame deletion (ΔGCGCAC) within the annotated gene phenylalanine ammonia-lyase 1 (*Fvpal1*). This mutation was found to cause a deletion, without a frameshift, of two amino acids at positions 112 and 113 (arginine and threonine, respectively) in the *Fvpal1* gene of the white strain. Specific primers to detect this mutation were designed, and amplification refractory mutation system (ARMS) polymerase chain reaction (PCR) was performed to evaluate whether the mutation is color specific for the *F. velutipes* fruiting body. PCR analysis of a total of 95 *F. velutipes* strains revealed that this mutation was present only in white strains. In addition, monospores of the heterozygous mutant were isolated, and whether this mutation was related to the color of the fruiting body was evaluated by a mating assay. In the mating analysis of monospores with mutations in *Fvpal1*, it was found that this mutation plays an important role in determining the color of the fruiting body. Furthermore, the deletion (Δ^112^RT^113^) in *Fvpal1* is located between motifs that play a key role in the catalytic function of FvPAL1. These results suggest that this mutation can be used as an effective marker for the color-specific breeding of *F. velutipes*, a representative edible mushroom.

## 1. Introduction

**Figure 1:**
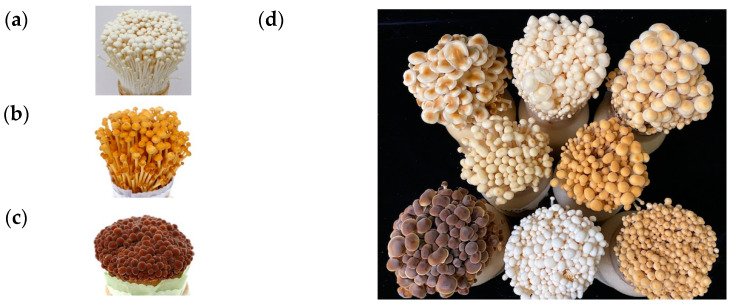
Color of the fruiting bodies of *Flammulina velutipes* strains. (**a**) *F. velutipes* white strain; (**b**) *F. velutipes* yellowish-brown strain; (**c**) *F. velutipes* dark-brown strain; (**d**) various colors of *F. velutipes* strains.

The edible mushroom *Flammulina velutipes* belongs to the family Tricholomataceae within Agaricales and grows on old trees or the stumps of various broadleaf trees from late autumn to the following spring. This mushroom is a cold-resistant fungus that occurs even in winter; therefore, it is also called the winter mushroom [1,2]. Wild-type *F. velutipes* strains vary in color from yellowish-brown to dark brown, whereas artificial cultivars are predominantly white [3] (Figure 1). The first artificially cultivated variety was a brown *F. velutipes* strain, but the cultivation of *F. velutipes* began in earnest after white varieties were bred in Japan, and these are still the main cultivated varieties today [3,4]. The white *F. velutipes* mainly cultivated in Korea and Japan were discovered by chance, and the genetic cause of the discoloration of the fruiting body is not known. Since the artificial cultivation of *F. velutipes* began, various white cultivars have been actively developed, but the close genetic relationship between these varieties limits the development of new cultivars [3,4]. To overcome these limitations, new varieties are being actively developed through crossbreeding with various wild strains, including non-white ones [3,5,6,7,8,9,10,11]. Recent studies have analyzed nutritional components, such as saccharides, amino acids, and organic acids, as well as the growth characteristics of new varieties, and a comparative analysis of nutrients between white and non-white varieties was also conducted [3,5,6,7,8,9,10,11,12,13,14,15,16,17]. Information on the physiological characteristics and differences of these *F. velutipes* varieties, including the color of the *F. velutipes* fruiting body, can be used for breeding newly developed cultivars. While research on the development of non-white *F. velutipes* varieties is being conducted, studies have also been carried out to suppress the browning of mushrooms. Browning of *Agaricus bisporus* (button mushrooms) is a serious problem that shortens the shelf life after harvest. Mushroom browning is mainly caused by the activation of tyrosinase, belonging to the polyphenol oxidase (PPO) family, or spontaneous oxidation [18]. In mushrooms, it has been reported that the enzymatic oxidation of phenolic compounds leads to the biosynthesis of melanin, resulting in the formation of a brown color [19]. The key enzymes in the melanin biosynthesis pathway are PPO, laccase (EC 1.10.3.2), and tyrosinase (EC 1.14.18.1) and are involved in the conversion of phenolic compounds [20]. Tyrosinase converts monophenols to ortho-diphenols by ortho-hydroxylation, and ortho-diphenols to ortho-quinones by oxidation. Finally, quinone is converted to melanin via a non-enzymatic reaction. Laccases oxidize a variety of phenolic substrates by performing one-electron oxidation, leading to crosslinking and facilitating the biosynthesis of melanin pigments [21]. Another enzyme involved in browning is peroxidase, which catalyzes the oxidation of various compounds using hydrogen peroxide as a substrate [22,23]. Recently, the *PPO* gene relating to the color of the fruiting body of *Agaricus bisporus* was identified, and the development of a variety that suppresses browning through gene editing has been attempted [24]. The color of mushroom fruiting bodies is an important factor that affects not only the development of new varieties but also the mushroom industry.

As the genome sequences of *F. velutipes* species have been determined and in-depth genetic information has been revealed, comparative studies on the biological characteristics and diversity of these mushrooms have been actively conducted [1,25,26,27,28,29]. The genetic repertoire of *F. velutipes* mushrooms revealed through genomic studies has proven that this mushroom can be used for a wide variety of industrial applications and has ample potential for the development of new varieties. In this study, comparative genome analysis of *F. velutipes* was performed to identify key genes or mutations related to the color of the *F. velutipes* fruiting body. The color-related genes or mutations of *F. velutipes* identified in this study can be used to selectively develop the color of this mushroom variety in the future.

## 2. Materials and Methods

### 2.1. Fungal Strain Culture and Genomic DNA Isolation

*F. velutipes* strains (Table 1) were obtained from the Mushroom Research Division, National Institute of Horticultural and Herbal Science (Rural Development Administration, Jeonju, Republic of Korea) and were grown on potato dextrose agar (PDA; 4 g potato starch, 20 g dextrose, 15 g agar per liter) at 25 °C for 15 days. Genomic DNA was then extracted using extraction buffer (0.25 M Tris-HCl, 100 mM NaCl, 50 mM ethylenediaminetetraacetic acid, 5% SDS), 2 × CTAB buffer (100 mM Tris-HCl pH 8, 20 mM EDTA pH 8, 2% CTAB, 1.4 M NaCl, and 1% polyvinyl pyrrolidone), and phenol-chloroform-isoamyl alcohol (25:24:1) as previously described [29]. The extracted DNA samples were treated with RNase A (Qiagen, Hilden, Germany).

### 2.2. Genome Sequencing and Identification of Variations

Genome sequencing of *F. velutipes* strains was performed using the HiSeq 2000 platform (Illumina, Inc., San Diego, CA, USA). Sequenced reads were processed using FastQC (http://www.bioinformatics.babraham.ac.uk/projects/fastqc/, accessed on 11 August 2022) and Trimmomatic (version 0.39) [30] for quality control. The final reads were used for mapping to the reference genome (*F. velutipes* KACC42780; accession number) [1] and to identify sequence variations using the genome analysis toolkit (GATK) pipeline [31] with the Burrows–Wheeler Aligner (BWA) [32], SAMtools [33], and PICARD [34]. SnpEff [35] and BEDTools [36] were used to identify the locations of variations within the genes. Gene modeling of the reference genome was performed by the Funannotate pipelines [37], using *F. velutipes* KACC43778 transcriptome data as “evidence”. Gene models of *F. velutipes* KACC42780 were annotated using KEGG, InterPro, and UniProt databases. Genome sequencing reads were deposited in NABIC (National Agricultural Biotechnology Information Center, RDA, Korea) (see Data Availability Statement)**.**

### 2.3. Primer Design and Amplification Refractory Mutation System PCRs

Primers that amplified specific variation sites within the phenylalanine ammonia-lyase 1 (*Fvpal1*) gene of white and non-white *F. velutipes* strains were designed based on the amplification refractory mutation system (ARMS) [38]. Three primer sets were designed with expected amplicons of 464 bp, 293 bp, and 200 bp from all *F. velutipes* strains, *F. velutipes* white strains, and *F. velutipes* non-white strains, respectively (Table 2). Genomic DNA was used as a template (100 ng/μL) for ARMS PCR reactions using the Taq PreMix kit (TNT Research, Anyang, Korea) and 0.25 pmol of each primer in a 20 μL reaction mixture. PCR conditions were 10 min of initial denaturation at 94 °C, followed by 30 cycles of denaturation at 94 °C for 30 s, annealing at 56 °C for 30 s, extension at 72 °C for 1 min, and a final extension at 72 °C for 10 min using Bio-Rad thermal cycler (Bio-Rad Laboratories Inc., Hercules, CA, USA). The amplified products were separated on a 2% agarose gel in 0.5 × TAE buffer (Tris–acetic acid–EDTA, TNT Research, Anyang, Korea) buffer and visualized with ethidium bromide on a UV transilluminator.

### 2.4. Mating of F. velutipes Strains

For spore collection of *F. velutipes* ASI4175 (non-white, heterozygote for ΔGCGCAC *Fvpal1*) and *F. velutipes* w1-8 (white, homozygote for ΔGCGCAC *Fvpal1*) strains, the stipe of the fruiting body was removed, and only part of the pileus was separated and placed in a Petri dish with the fold of the pileus facing down, and spores were collected for 24 h. The collected spores were diluted to a concentration of 1.0 × 10^−3^ cfu/mL, and 100 μL of this dilution was spread on PDA medium and cultured for 7 d in a 25 °C incubator, protected from light. The germinated spores were inoculated into PDA medium and further cultured for 7 d in an incubator at 25 °C. Monokaryons without clamp connection were selected among the cultured mycelia. The *Fvpal1* genotype of the isolated monokaryons was analyzed by ARMS PCR to select those for mating.

The monokaryons isolated from *F. velutipes* ASI4175 and *F. velutipes* w1-8 were inoculated on PDA medium at intervals of 1–2 cm and cultured for 7 d in a 25 °C incubator; hybrid dikaryons with clamp connection were then selected for fruiting. The hybrid dikaryons were inoculated into fruiting medium (80% sawdust and 20% rice bran) and incubated at 20 °C and 65% humidity for 30 days; the temperature was then sequentially changed from 14 °C (95% humidity) to 7 °C (80% humidity) for fruiting.

## 3. Results and Discussion

### 3.1. Identification of Fruiting Body Color-Specific Mutation of F. velutipes

The quality-trimmed reads of the six *F. velutipes* genome sequences were mapped to the reference genome sequence (*F. velutipes* KACC42870) at a rate of 73.15–80.13% (Table 3).

**Figure 2:**
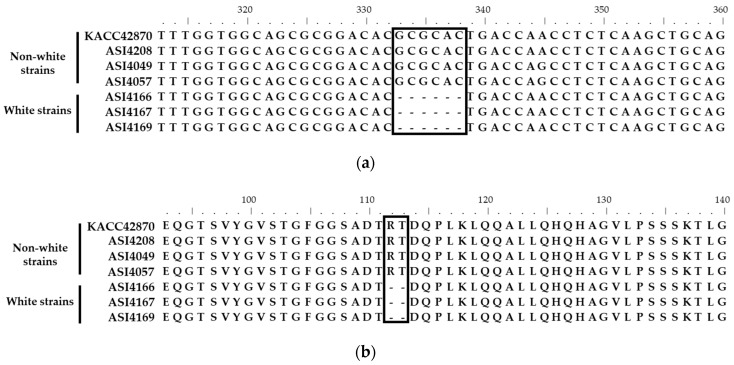
Alignments of phenylalanine ammonia-lyase 1 (*Fvpal1*) genes of *Flammulina velutipes* strains. (**a**) cDNA sequences; (**b**) amino acid sequences. Boxes indicate the variation sites of the *Fvpal1* gene. The white strains of *F. velutipes* showed a deletion of GCGCAC (^112^RT^113^; arginine and threonine) in the *Fvpal1* gene.

A total of 70 white-color-specific variations, including 57 single nucleotide polymorphisms (SNPs) and 13 indels, were identified from the genome comparison of non-white and white *F. velutipes* strains (Table S1). Among the 11 chromosomes of *F. velutipes*, chromosome 7 had the highest number of variations (60%, 33 SNPs, and 9 indels), suggesting that genetic variations among *F. velutipes* strains mainly occurred on chromosome 7. Gene modeling of the reference strain (KACC42870) was conducted to identify variations in the genes. Using transcriptome data from Funannotate pipelines, a total of 15,874 gene models were identified from the reference genome (KACC42870) (Table S2). Gene annotations revealed that 36 predicted genes were associated with the identified variations (Table S1). Among the variations in the genes, only one variation (indel) in *Fvpal1* was identified; with a disruptive in-frame deletion in the exon of white *F. velutipes* strains. This variation was caused by a six-nucleotide (GCGCAC) deletion in the *Fvpal1* gene of the white *F. velutipes* strains (Figure 2 and Figure S1). The six-nucleotide deletion was located in the third exon of the *Fvpal1* gene and resulted in arginine and threonine deletions without frameshift or reading frame interruption.

### 3.2. Primers Design and ARMS PCR for Fruiting Body Color Discrimination of F. velutipes

**Figure 3:**
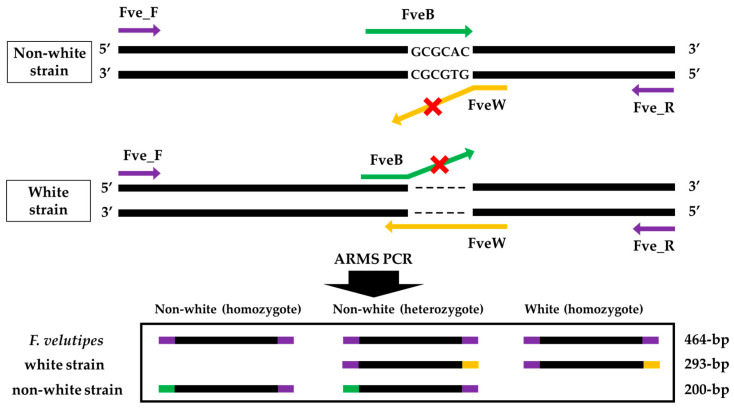
Primer design for non-white or white *Flammulina velutipes* strain discrimination by amplification refractory mutation system (ARMS) PCR. Primers were designed to amplify the *Fvpal1* gene with 464 bp and 200 bp, 464 bp and 293 bp from non-white and white strain, respectively. A heterozygote of the *Fvpal1* gene would be expected to show all three bands.

Figure 3 shows the scheme of ARMS PCR primer design to detect the specific variation ΔGCGCAC in the *Fvpal1* gene and to discriminate the fruiting body color of *F. velutipes*. A four-primer set was designed to amplify all *F. velutipes*, non-white, or white strains (Table 2). The FveF and FveR primers were expected to amplify the 464 bp product from all *F. velutipes* strains. In addition, the FveF/FveW and FveR/FveB primer sets were expected to amplify 293 bp and 200 bp products for *F. velutipes* white and non-white strains, respectively.

**Figure 4:**
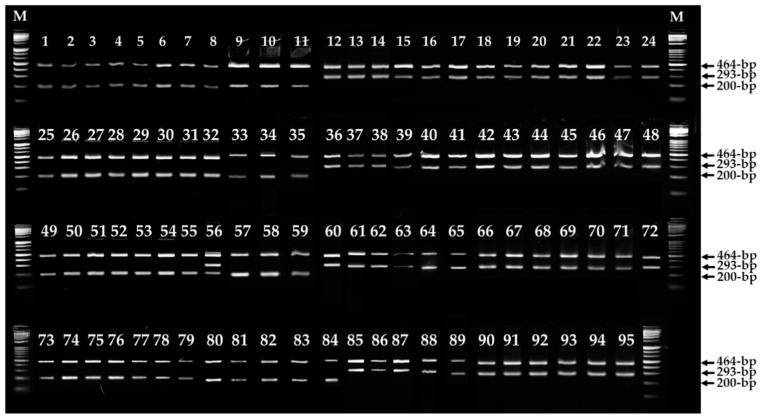
Amplification refractory mutation system (ARMS) PCR of *Fvpal1* in 95 *Flammulina velutipes* strains (Table 1). Non-white and white strains of *F. velutipes* showed 464 bp and 200 bp, 464 bp and 293 bp PCR products, respectively. A heterozygous strain (No. 56, *F. velutipes* ASI4175) showed all three amplification products: 464 bp, 293 bp, and 200 bp. M, size marker (1 kb ladder, TNT Research).

ARMS is a simple and reliable method for identifying single nucleotide variations (SNVs) or deletions [38]. Since ARMS uses PCR primers that allow the amplification of DNA only in the presence of specific mutations, the amplification of ARMS PCR products determines the presence of mutations. As shown in Figure 4, ARMS PCR analysis revealed the specific detection of the ΔGCGCAC variation in *Fvpal1,* as well as the specific discrimination of non-white and white *F. velutipes* strains. Among the 95 *F. velutipes* strains tested, ARMS PCR amplified 464 bp and 293 bp products in all white strains. The non-white strains amplified either 464 bp and 200 bp or 464 bp, 200 bp, and 293 bp products. The *F. velutipes* ASI4175 non-white strain amplified all three products, indicating that it was heterozygous for the normal and ΔGCGCAC *Fvpal1* gene. These results suggest that the normal *Fvpal1* gene has a dominant effect on the fruiting body color of *F. velutipes*, as *F. velutipes* ASI4175 is a non-white strain. In this study, *Fvpal1* gene mutations in 50 white strains were detected using specific primers and ARMS PCR analysis, and as a result, the fruiting body color of *F. velutipes* strains was accurately discriminated.

Although the color of the fruiting body was accurately discriminated by analysis of the 95 strains used in this study, future research should continuously analyze an additional *F. velutipes* strain to determine whether the variation in *Fvpal1* affects the color of the fruiting body.

### 3.3. Mutation in the Fvpal1 Gene Affect Fruiting Body Color of F. velutipes

Phenylalanine ammonia-lyase (PAL; EC 4.3.1.24) catalyzes the deamination of l-phenylalanine to trans-cinnamic acid and is commonly found in plants and fungi [39,40]. PAL is involved in the first step of the phenylpropanoid pathway, leading to the synthesis of various phenylpropanoids such as flavonoids, isoflavonoids, anthocyanins, lignins, and other phenolic compounds [40]. Therefore, PAL is considered to be a key initiator of the phenylpropanoid pathway, a transition process from primary to secondary metabolism.

In plants, particularly in lettuce, 5-caffeoylquinic acid, 3,5-dicaffeoylquinic acid, caffeoyltartaric acid, and dicaffeoyltartaric acid have been reported to be associated with browning [41,42]. It has also been reported that the phenylpropanoid pathway is activated by wounds or hormones, such as ethylene, to increase the synthesis of phenolic compounds [41,43]. Therefore, PAL has been extensively studied to increase the understanding of metabolic processes as well as browning. It has also been reported that PAL enzyme and phenolic compounds are essential for the browning of mung bean sprouts [44]. In addition, phenolic compounds, including *trans*-caffeoyltartronic acid and *trans*-coumaroyltartronic acid, are substrates of polyphenol oxidase (PPO; EC 1.10.3.1) in mung bean sprouts and act as major factors for browning [44]. It has been suggested that the polyphenols in mung bean sprouts increase gradually during storage and are oxidized by PPO to form a brown pigment [45,46].

It has been reported that the fungal PAL enzyme degrades phenylalanine via a pathway similar to that in plants [47,48]. Further research revealed the phenylalanine metabolic pathway in some basidiomycetes, including *Rhodotorula glutinis*, *Schizophyllum commune*, and *Sporobolomyces roseus* [48,49,50]. In the phytopathogenic fungi *Moniliophthora perniciosa,* PAL has been found to accumulate during the infection stage, suggesting that it may be associated with pathogenicity [51]. Additionally, *Tricholoma matsutake* and *F. velutipes* PAL mRNAs were expressed specifically at the developmental stage, and in *F. velutipes*, the highest expression was found in the mycelium and when l-tyrosine was added [52,53]. Although there have been reports of various roles for PAL, it has not been reported that it is associated with the color of fungi, including mushrooms.

**Figure 5:**
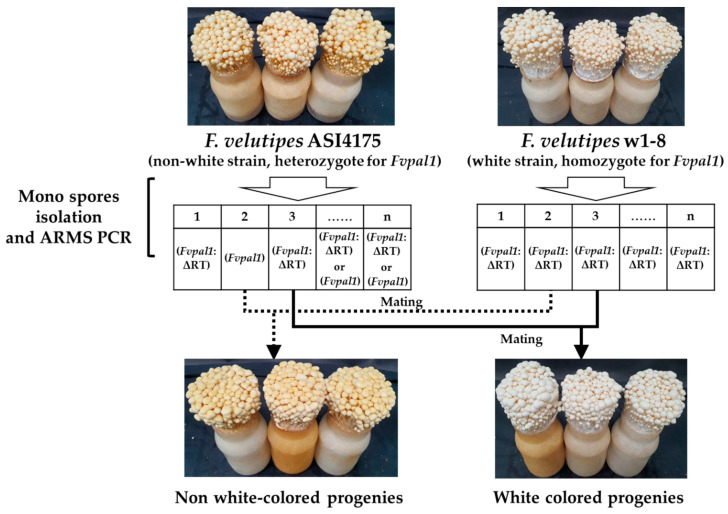
Matings of monospores from *Flammulina velutipes* strains ASI4175 (non-white, heterozygote) and w1-8 (white, homozygote). The isolated monospores were analyzed for the deletion of arginine and threonine; Δ^112^RT^113^ in the *Fvpal1* gene using ARMS PCR. The number in the box indicates the isolated monospore.

Sixty monospores were isolated from each of *F. velutipes* ASI4175 (non-white, heterozygote for ΔGCGCAC *Fvpal1*) and w1-8 (white, homozygote for ΔGCGCAC *Fvpal1*), based on the ARMS PCR results (Figure 4). Each monospore was evaluated for mutations (ΔGCGCAC) in the *Fvpal1* gene through ARMS PCR analysis, and monospores containing 15 mutated *Fvpal1* (ΔGCGCAC) genes and 15 non-mutated *Fvpal1* genes were obtained from *F. velutipes* ASI4175 (Figure S2). As shown in Figure 5, progenies from the mating of monospores with the ΔGCGCAC deletion and monospores without the ΔGCGCAC deletion in the *Fvpal1* gene showed non-white fruiting bodies. However, the mating of monospores containing the mutated *Fvpal1* gene (ΔGCGCAC) isolated from *F. velutipes* ASI4175 (non-white, heterozygote for ΔGCGCAC *Fvpal1*) and w1-8 (white, homozygote for ΔGCGCAC *Fvpal1*) produced progenies with only white-colored fruiting bodies.

In this study, mating analysis revealed that the *Fvpal1* gene plays an important role in the fruiting body color of *F. velutipes*, suggesting that this gene could be a useful marker for the selective breeding of new varieties of *F. velutipes*, especially for fruiting body color.

### 3.4. Structural Characteristics of the PAL of F. velutipes

**Figure 6:**
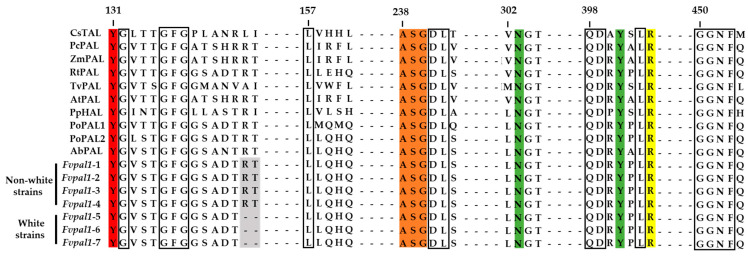
Alignment of the highly conserved domains of phenylalanine/tyrosine/histidine ammonia-lyase amino acids. Red: catalytically essential tyrosine residue, orange: the MIO forming amino acid triad, green: amino acid stabilizing MIO group, yellow: arginine responsible for binding the carboxylic group of the substrate, gray: variation site (arginine and threonine) of *F. velutipes*, boxes: other conserved catalytic and binding residues. *Cereibacter sphaeroides* TAL (CsTAL, UniProtKB ID; Q3IWB0), *Petroselinum crispum* PAL (PcPAL, UniProtKB ID; P24481), *Zea mays* PAL (ZmPAL, UniProtKB ID; C0HJ40), *Rhodosporidium toruloides* PAL (RtPAL, UniProtKB ID; P11544), *Trichormus variabilis* PAL (TvPAL, UniProtKB ID; Q3M5Z3), *Arabidopsis thaliana* PAL (AtPAL, UniProtKB ID; P45724), *Pseudomonas putida* HAL (PpHAL, UniProtKB ID; Q88CZ7), *Pleurotus ostreatus Fvpal1* (PoPAL1, UniProtKB ID; A0A4Y6HUI7), *Pleurotus ostreatus* PAL2 (PoPAL2, UniProtKB ID; A0A4Y6HUD7), *Agaricus bisporus* PAL (AbPAL, KEGG ID; T03136), *F. velutipes* KACC42870 (non-white strain) *Fvpal1* (*Fvpal1*-1), *F. velutipes* ASI4028 (non-white strain) *Fvpal1* (*Fvpal1*-2), *F. velutipes* ASI4049 (non-white strain) *Fvpal1* (*Fvpal1*-3), *F. velutipes* ASI4057 (non-white strain) *Fvpal1* (*Fvpal1*-4), *F. velutipes* ASI4166 (white strain) *Fvpal1* (*Fvpal1*-5), *F. velutipes* ASI4157 (white strain) *Fvpal1* (*Fvpal1*-6), *F. velutipes* ASI4169 (white strain) *Fvpal1* (*Fvpal1*-7).

The catalytic prosthetic 3,5-dihydro-5-methylidine-4*H*-imidazol-4-one group (MIO) is essential for the catalytic activity of PAL and is produced by the autocatalytic crystallization of amino acids, including alanine, serine, and glycine [40,54,55]. The MIO group is commonly found in ammonia lyases, including PAL, tyrosine ammonia lyase (TAL), and histidine ammonia lyase (HAL), and the ASG motif plays an important role in this enzymatic activity [56,57,58,59]. A highly conserved MIO group (ASG motif) was also found at positions 238–240 in *Fvpal1* of *F. velutipes* in both the white and non-white strains (Figure 6). Another highly conserved motif was also found in *Fvpal1* of *F. velutipes*, including stabilizing residues for the MIO group (N^303^ and Y^401^) and carboxylic-acid-binding residues for the substrate (R^404^).

Among the PAL and TAL motifs, specific residues for substrate specificity are phenylalanine-leucine (FL) for phenylalanine and histidine-leucine (HL) for tyrosine. A characteristic histidine-glycine (HQ) motif of the PAL enzyme, which exhibits substrate activity for both tyrosine and phenylalanine, has also been reported [60,61]. For PAL and TAL enzymes with an HQ motif, it has been reported to have dual substrate activity with a *K*_*m*Phenylalanine/Tyrosine_ ratio greater than one [61,62,63,64]. A histidine-glycine (^160^HQ^161^) motif was also found in the FvPAL1 of *F. velutipes* as well as other species, including *P. ostreatus* and *A. bisporus* (Figure 6). Although specific activity assays for phenylalanine and tyrosine are required, the HQ motif in the FvPAL1 of *F. velutipes* suggests that this enzyme could possibly catalyze both substrates.

**Figure 7:**
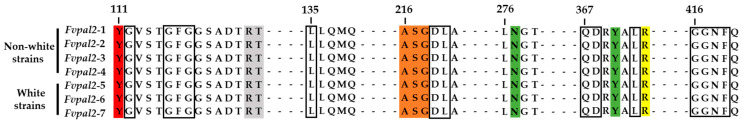
Alignment of the highly conserved domains of phenylalanine ammonia-lyase 2 amino acids of *Flammulina velutipes* strains. Red: catalytically essential tyrosine residue, orange: the MIO forming amino acid triad, green: amino acid stabilizing MIO group, yellow: arginine responsible for binding the carboxylic group of the substrate, gray: variation site of *Fvpal1* gene of *F. velutipes*, boxes: other conserved catalytic and binding residues. *F. velutipes* KACC42870 (non-white strain) *Fvpal2* (*Fvpal2*-1), *F. velutipes* ASI4028 (non-white strain) *Fvpal2* (*Fvpal2*-2), *F. velutipes* ASI4049 (non-white strain) *Fvpal2* (*Fvpal2*-3), *F. velutipes* ASI4057 (non-white strain) *Fvpal2* (*Fvpal2*-4), *F. velutipes* ASI4166 (white strain) *Fvpal2* (*Fvpal2*-5), *F. velutipes* ASI4157 (white strain) *Fvpal2* (*Fvpal2*-6), *F. velutipes* ASI4169 (white strain) *Fvpal2* (*Fvpal2*-7).

In this study, two PAL genes, *Fvpal1* and *Fvpal2*, were identified in the genome of *F. velutipes*. No mutations were found in the sequence of the *Fvpal2* gene (Figure S3). As shown in Figure 7, the fruiting body color-related mutation Δ^112^RT^113^ was not found in the *Fvpal2* gene of either non-white or white *F. velutipes* strains. Motifs essential for the catalytic activity of PAL enzymes were found in *Fvpal2* genes. However, substrate-specific residues of *Fvpal2* (^138^MQ^139^) were found to be different from those of the *Fvpal1* gene (^160^HQ^161^) but identical to those of the PoPAL1 gene (^160^MQ^161^) of *P. ostreatus* (Figure 6 and Figure 7).

In a previous study [53], *Fvpal* was identified in *F. velutipes* and was found to have the same sequence as the *Fvpal1* gene of the *F. velutipes* non-white strain identified in this study, which consisted of 2746 bp and 12 exons and showed 100% identity to *Fvpal,* with 724 amino acids (2175 bp cDNA). However, Δ^112^RT^113^ deletions identified in the *Fvpal1* gene were not found in the *Fvpal* gene sequence. These results indicate that the previously reported *Fvpal* gene [53] was identified in non-white *F. velutipes,* and this was confirmed with the information that the non-white strain *F. velutipes* 4164 was used for the identification of the *Fvpal* gene [53]. The *Fvpal2* gene is 2617 bp in size with eight exons and consists of a 2208 bp cDNA that is translated into 735 amino acids (Figure S3). Furthermore, among the *Fvpal2* genes identified from *F. velutipes* strains, no mutations specific to non-white or white strains were found, except for variations for each strain. Although further studies are required, these results suggest that the *Fvpal2* gene is essential for the physiological and metabolic functions of *F. velutipes* but that the *Fvpal1* gene has the potential to function selectively in determining the color of the *F. velutipes* fruiting body.

## 4. Conclusions

In nature, *Flammulina velutipes* forms non-white fruiting bodies, but white fruiting body varieties were accidentally developed by artificial breeding. However, until recently the physiological, biochemical, and genetic causes associated with the formation of white fruiting bodies in *F. velutipes* had not been elucidated. A recent comparative analysis reported that components of non-white *F. velutipes* strains, such as amino acids, saccharides, and β-glucan, were relatively higher or lower than those of white strains [6,12,13,14,15,16,17]. These results suggest that the fruiting body color of *F. velutipes* can be used as a criterion for breeding new varieties. Therefore, the selective breeding of fruiting body color is considered a great advantage for efficient breeding.

In this study, comparative genomics of six *F. velutipes* strains showed 70 variations unique to white strains, including SNPs and indels. Of these, 36 were found in open reading frames and only one caused a disruptive in-frame deletion (ΔGCGCAC) in the *Fvpal1* gene, resulting in the deletion of two amino acids (arginine and threonine) at positions 112 and 113. Specific primers were designed to detect this mutation, and PCR analysis of 95 *F. velutipes* strains revealed that this mutation was present only in white strains. In addition, monospores of the heterozygous mutant were isolated, and a mating assay was performed to evaluate the mutation’s relationship to fruiting body color. As a result, progeny resulting from mating monospores with and without the ΔGCGCAC deletion in the *Fvpal1* gene showed non-white fruiting bodies. However, mating monospores with the mutated *Fvpal1* gene resulted in progeny with only white-colored fruiting bodies.

Although the effect of mutations in the *Fvpal1* gene of *F. velutipes* on enzyme activity and metabolic function remains to be studied, it is considered that this gene can be effectively used for selective breeding of this mushroom.

## Figures and Tables

**Table 1:** *Flammulina velutipes* strains used in this study.

No.	Strains	Color	No.	Strains	Color	No.	Strains	Color
1	ASI4019	non-white	41	T011	white	81	B21	non-white
2	ASI4019-20	non-white	42	honam	white	82	B8	non-white
3	ASI4019-1820	non-white	43	daeheung	white	83	B26	non-white
4	ASI4028	non-white	44	W6-8	white	84	B39	non-white
5	ASI4049	non-white	45	W6-18	white	85	baegi	white
6	ASI4019-2003	non-white	46	W6-19	white	86	baekseung	white
7	ASI4057	non-white	47	W6-14	white	87	baekjung	white
8	ASI4064	non-white	48	W1-9	white	88	7937	white
9	ASI4067	non-white	49	hwanggeum	non-white	89	8492	white
10	ASI4069	non-white	50	B151	non-white	90	naju	white
11	ASI4072	non-white	51	B129	non-white	91	ASI4100	white
12	ASI4074	white	52	B112	non-white	92	ASI4102	white
13	ASI4166	white	53	B13	non-white	93	hampyeong	white
14	ASI4167	white	54	yeoreumhyang2ho	non-white	94	W8-17	white
15	ASI4168	white	55	B16	non-white	95	ASI4168	white
16	ASI4169	white	56	4175	non-white			
17	ASI4178	white	57	B17	non-white			
18	ASI4029	white	58	B18	non-white			
19	ASI4210	white	59	B62	non-white			
20	ASI4216	white	60	W5-2	white			
21	ASI4217	white	61	W1-18	white			
22	ASI4226	white	62	W6-13	white			
23	ASI4228	white	63	W8-18	white			
24	ASI4230	white	64	W1-23	white			
25	ASI4083	non-white	65	W1-8	white			
26	ASI4090	non-white	66	W3-24	white			
27	ASI4103	non-white	67	W4-16	white			
28	ASI4111	non-white	68	W5-3	white			
29	ASI4146	non-white	69	W5-16	white			
30	ASI4148	non-white	70	W6-13	white			
31	ASI4149	non-white	71	W6-16	white			
32	ASI4163	non-white	72	W8-1	white			
33	ASI4218	non-white	73	B70	non-white			
34	ASI4219	non-white	74	B74	non-white			
35	ASI4232	non-white	75	B87	non-white			
36	ASI4231	white	76	B66	non-white			
37	ASI0003	white	77	B127	non-white			
38	ASI0019	white	78	B162	non-white			
39	jeonnam	white	79	B63	non-white			
40	cheongdo	white	80	B121	non-white			

**Table 2:** Primers used in this study.

Primer	Sequences (5′–3′)	Target	Amplicon Size (Base Pair)
Fve_F	TCTCCACTTACCTTCTCCTA	*Flammulina velutipes* strains	464
Fve_R	TATGGTAAGTACACGGTCAG		
Fve_F	TCTCCACTTACCTTCTCCTA	*Flammulina velutipes* white strains	293
FveW	TTGAGAGGTTGGTCAGTGTC		
Fve_R	TATGGTAAGTACACGGTCAG	*Flammulina velutipes* non-white strains	200
FveB	TTCCTAGCGGACACGCGCAC		

**Table 3:** List of *Flammulina velutipes* strains and genome sequencing statics.

Strains	Fruiting Body Color	Sequencing Reads		Mapping Rate (%)	References or Accession Number
		Total Reads	Trimmed Reads (%)		
*F. velutipes* KACC42870	non-white	Reference strain			[1]
*F. velutipes* KACC43778	non-white	Reference strain			[1]
*F. velutipes* ASI4028	non-white	7,510,278	6,355,902 (84.63)	77.93	NN-1534-000001
*F. velutipes* ASI4049	non-white	11,535,948	9,447,436 (81.9)	80.13	NN-1535-000001
*F. velutipes* ASI4057	non-white	8,134,556	6,949,358 (85.43)	77.58	NN-1537-000001
*F. velutipes* ASI4166	white	29,145,728	26,852,658 (92.13)	73.15	NN-0798-000001
*F. velutipes* ASI4167	white	13,665,794	11,211,378 (82.04)	75.89	NN-1545-000001
*F. velutipes* ASI4169	white	17,619,236	14,367,908 (81.55)	78.61	NN-1546-000001

## Data Availability

NGS reads were deposited in the NABIC (National Agricultural Biotechnology Information Center, RDA, Korea; https://nabic.rda.go.kr/, accessed on 11 August 2022) Sequence Read Archive (SRA) (Table 3).

## Supplementary Materials

The following supporting information can be downloaded at https://www.mdpi.com/article/10.3390/jof9030339/s1: Figure S1: Alignments of phenylalanine ammonia-lyase 1 (*Fvpal1*) genes in *Flammulina velutipes* strains. (a) cDNA sequence and (b) amino acid sequence. *F. velutipes* KACC42870 (non-white), *F. velutipes* ASI4208 (non-white), *F. velutipes* ASI4049 (non-white), *F. velutipes* ASI4057 (non-white), *F. velutipes* ASI4166 (white), *F. velutipes* ASI4167 (white), *F. velutipes* ASI4169 (white). Figure S2: Amplification refractory mutation system (ARMS) PCR of *Fvpal1* in monospores from *Flammulina velutipes* ASI4175 (heterozygote, non-white) (a) and w1-8 (homozygote, white) (b) strains. Asterisks indicate *F. velutipes* monospores without mutation in the *Fvpal1* gene. Figure S3: Alignments of phenylalanine ammonia-lyase 2 (*Fvpal2*) genes of *Flammulina velutipes* strains. (a) cDNA sequence and (b) amino acid sequence. *F. velutipes* KACC42870 (non-white), *F. velutipes* ASI4208 (non-white), *F. velutipes* ASI4049 (non-white), *F. velutipes* ASI4057 (non-white), *F. velutipes* ASI4166 (white), *F. velutipes* ASI4167 (white), *and F. velutipes* ASI4169 (white). Table S1: White-color-specific variations identified in *Flammulina velutipes* strains. Table S2: Predicted gene models of *Flammulina velutipes* KACC42780.
