# Supplementary material for: Phenylalanine Ammonia-Lyase: A Key Gene for Color Discrimination of Edible Mushroom Flammulina velutipes
Source: J Fungi (Basel). 2023 Mar 9;9(3):339. doi: 10.3390/jof9030339 (PMC10053379; doi:10.3390/jof9030339)
Supplement: Supplementary file 1 [file jof-09-00339-s001.zip › Figure S2.pdf]

```
                 10        20        30        40        50        60        70
KACC42870  ATGGCCATCTTATCTGAGACTCGTACCGTCCGTCGCACCTCTTCTGCCCCCGCTCCCGCGGCATCACTAC
ASI4208    ATGGCCATCTTATCTGAGACTCGTACCGTCCGCCGCACCTCTTCTGCCCCCGCTCCCGCGGCATCACTAC
ASI4049    ATGGCCATCTTATCTGAGACTCGTACCGTCCGCCGCACCTCTTCTGCCCCCGCTCCCGCGGCATCACTAC
ASI4057    ATGGCCATCTTATCTGAGACTCGTACCGTCCGCCGCACCTCTTCTGCCCCCGCTCCCGCGGCATCACTAC
ASI4166    ATGGCCATCTTATCTGAGACTCGTACCGTCCGCCGCACCTCTTCTGCCCCCGCTCCCGCGGCATCACTAC
ASI4167    ATGGCCATCTTATCTGAGACTCGTACCGTCCGCCGCACCTCTTCTGCCCCCGCTCCCGCGGCATCACTAC
ASI4169    ATGGCCATCTTATCTGAGACTCGTACCGTCCGCCGCACCTCTTCTGCCCCCGCTCCCGCGGCATCACTAC

                 80        90       100       110       120       130       140
KACC42870  TCGAACGAAAAGACCCAGCGTCTTATACAACACATTCGGCAAAACTGCTAGAAAAGTTCGTTGCGTCCTA
ASI4208    TCGAACGAAAAGACCCAGCGTCTTATACAACACATTCGGCAAAACTGCTAGAAAAGTTTGTTGCGTCCTA
ASI4049    TCGAACGAAAAGACCCAGCGTCTTATACAACACATTCGGCCAAACTGCTAGAAAAGTTTGTTGCGTCCTA
ASI4057    TCGAACGAAAAGACCCAGCGTCTTATACAACACATTCGGCAAAACTGCTAGAAAAGTTTGTTGCGTCCTA
ASI4166    TCGAACGAAAAGACCCAGCGTCTTATACAACACATTCGGCAAAACTGCTAGAAAAGTTTGTTGCGTCCTA
ASI4167    TCGAACGAAAAGACCCAGCGTCTTATACAACACATTCGGCAAAACTGCTAGAAAAGTTTGTTGCGTCCTA
ASI4169    TCGAACGAAAAGACCCAGCGTCTTATACAACACATTCGGCAAAACTGCTAGAAAAGTTTGTTGCGTCCTA

                150       160       170       180       190       200       210
KACC42870  CAGAGAGCTCGAGTCCTACAAGCATGGCAAGACTGTTCACGTTGATGGACAGACGCTATCGATTGCTGCC
ASI4208    CAGAGAGCTCGAGTCCTACAAGCATGGCAAGACTGTTCACGTTGATGGACAGACACTATCGATCGCTGCC
ASI4049    CAGAGAGCTCGAGTCCTACAAGCATGGCAAGACTGTTCACGTTGATGGACAGACACTATCGATCGCTGCC
ASI4057    CAGAGAGCTCGAGTCCTACAAGCATGGCAAGACTGTTCACGTTGATGGACAGACGCTATCGATTGCTGCC
ASI4166    CAGAGAGCTCGAGTCCTACAAGCATGGCAAGACTGTTCACGTTGATGGACAGACACTATCGATCGCTGCC
ASI4167    CAGAGAGCTCGAGTCCTACAAGCATGGCAAGACTGTTCACGTTGATGGACAGACACTATCGATCGCTGCC
ASI4169    CAGAGAGCTCGAGTCCTACAAGCATGGCAAGACTGTTCACGTTGATGGACAGACACTATCGATCGCTGCC

                220       230       240       250       260       270       280
KACC42870  GTCACAGCTGCTGCGCGCTACGACACTCCAGTTCACCTCACCAGCTCTCAGGACGTGAAAAAACGCGTCG
ASI4208    GTCACAGCTGCTGCGCGCTACGACACTCCAGTTCACCTCACCAGCTCTCAGGACGTGAAAAAACGCGTCG
ASI4049    GTCACAGCTGCTGCGCGCTACGACACTCCAGTTCACCTCACCAGCTCTCAGGACGTGAAAAAACGCGTCG
ASI4057    GTCACAGCTGCTGCGCGCTACGACACTCCAGTTCACCTCACCAGCTCTCAGGACGTGAAAAAACGCGTCG
ASI4166    GTCACAGCTGCTGCGCGCTACGACACTCCAGTTCACCTCACCAGCTCTCAGGACGTGAAAAAACGCGTCG
ASI4167    GTCACAGCTGCTGCGCGCTACGACACTCCAGTTCACCTCACCAGCTCTCAGGACGTGAAAAAACGCGTCG
ASI4169    GTCACAGCTGCTGCGCGCTACGACACTCCAGTTCACCTCACCAGCTCTCAGGACGTGAAAAAACGCGTCG

                290       300       310       320       330       340       350
KACC42870  CGAAGAGTCGTTTAATTATCGCAGATAAGGTGGACAACGGCATCAGCGTCTATGGCGTTTCAACCGGGTT
ASI4208    CGAAGAGTCGTTTAATTATCGCAGATAAGGTGGACAACGGCATCAGCGTCTATGGTGTTTCAACCGGGTT
ASI4049    CGAAGAGTCGTTTAATTATCGCAGATAAGGTGGACAACGGCATCAGCGTCTATGGTGTTTCAACCGGGTT
ASI4057    CGAAGAGTCGTTTAATTATCGCAGATAAGGTGGACAACGGCATCAGCGTCTATGGCGTTTCAACCGGGTT
ASI4166    CGAAGAGTCGTTTAATTATCGCAGATAAGGTGGACAACGGCATCAGCGTCTATGGTGTTTCAACCGGGTT
ASI4167    CGAAGAGTCGTTTAATTATCGCAGATAAGGTGGACAACGGCATCAGCGTCTATGGTGTTTCAACCGGGTT
ASI4169    CGAAGAGTCGTTTAATTATCGCAGATAAGGTGGACAACGGCATCAGCGTCTATGGTGTTTCAACCGGGTT

                360       370       380       390       400       410       420
KACC42870  TGGAGGGAGTGCAGATACACGTACCGACCTTCCTATCGTTTTGGGGCATAGCTTGCTTCAGATGCAACAC
ASI4208    TGGAGGGAGTGCAGATACACGTACCGACCTTCCTATCGTTTTGGGGCATAGCTTGCTTCAGATGCAACAC
ASI4049    TGGAGGGAGTGCAGATACACGTACCGACCTTCCTATCGTTTTGGGGCATAGCTTGCTTCAGATGCAACAC
ASI4057    TGGAGGGAGTGCAGATACACGTACCGACCTTCCTATCGTTTTGGGGCATAGCTTGCTTCAGATGCAACAC
ASI4166    TGGAGGGAGTGCAGATACACGTACCGACCTTCCTATCGTTTTGGGGCATAGCTTGCTTCAGATGCAACAC
ASI4167    TGGAGGGAGTGCAGATACACGTACCGACCTTCCTATCGTTTTGGGGCATAGCTTGCTTCAGATGCAACAC
ASI4169    TGGAGGGAGTGCAGATACACGTACCGACCTTCCTATCGTTTTGGGGCATAGCTTGCTTCAGATGCAACAC

                430       440       450       460       470       480       490
KACC42870  AGTGGTGTCCTTCCATCTTCGACTCAGGCATTGGAAGCATTACCCCTCCTTGATCCAATGGGTTCGACTG
ASI4208    AGTGGTGTCCTTCCGTCTTCGACTCAGGCATTGGAAGCATTACCCCTCCTTGATCCAATGGGTTCGACTG
ASI4049    AGTGGTGTCCTTCCATCTTCGACTCAAGCATTGGAAGCGTTACCCCTCCTTGATCCAATGGGTTCGACTG
ASI4057    AGTGGTGTCCTTCCATCTTCGACTCAGGCATTGGAAGCATTACCCCTCCTTGATCCAATGGGTTCGACTG
ASI4166    AGTGGTGTCCTTCCGTCTTCGACTCAGGCATTGGAAGCATTACCCCTCCTTGATCCAATGGGTTCGACTG
ASI4167    AGTGGTGTCCTTCCGTCTTCGACTCAGGCATTGGAAGCATTACCCCTCCTTGATCCAATGGGTTCGACTG
ASI4169    AGTGGTGTCCTTCCGTCTTCGACTCAGGCATTGGAAGCATTACCCCTCCTTGATCCAATGGGTTCGACTG

                500       510       520       530       540       550       560
KACC42870  TGATGCCTGAATCTTGGGTTCGAGGGGCAATCCTCATCCGTATGAACTCACTTATCCGTGGTCACTCCGG
ASI4208    TGATGCCTGAATCTTGGGTTCGAGGGGCAATCCTCATCCGTATGAACTCACTTATCCGTGGTCACTCCGG
ASI4049    TGATGCCTGAATCTTGGGTTCGAGGGGCAATCCTCATCCGTATGAACTCACTTATCCGTGGTCACTCCGG
ASI4057    TGATGCCTGAATCTTGGGTTCGAGGGGCAATCCTCATCCGTATGAACTCACTTATCCGTGGTCACTCCGG
ASI4166    TGATGCCTGAATCTTGGGTTCGAGGGGCAATCCTCATCCGTATGAACTCACTTATCCGTGGTCACTCCGG
ASI4167    TGATGCCTGAATCTTGGGTTCGAGGGGCAATCCTCATCCGTATGAACTCACTTATCCGTGGTCACTCCGG
ASI4169    TGATGCCTGAATCTTGGGTTCGAGGGGCAATCCTCATCCGTATGAACTCACTTATCCGTGGTCACTCCGG
```

```
                     570       580       590       600       610       620       630
            ....|....|....|....|....|....|....|....|....|....|....|....|....|....|
KACC42870   AGTACGATGGGAGCTCATCGAAAAGATGAAGGCACTTCTTGACGCCAATATCACGCCCATCGTTCCTCTA
ASI4208     AGTACGATGGGAGCTCATCGAAAAGATGAAGGCACTTCTTGACGCCAATATCACGCCCATCGTTCCTCTA
ASI4049     AGTACGATGGGAGCTCATCGAAAAGATGAAGGCACTTCTTGACGCCAATATCACGCCCATCGTTCCTCTA
ASI4057     AGTACGATGGGAGCTCATCGAAAAGATGAAGGCACTTCTTGACGCCAATATCACGCCCATCGTTCCTCTA
ASI4166     AGTACGATGGGAGCTCATCGAAAAGATGAAGGCACTTCTTGACGCCAATATCACGCCCATCGTTCCTCTA
ASI4167     AGTACGATGGGAGCTCATCGAAAAGATGAAGGCACTTCTTGACGCCAATATCACGCCCATCGTTCCTCTA
ASI4169     AGTACGATGGGAGCTCATCGAAAAGATGAAGGCACTTCTTGACGCCAATATCACGCCCATCGTTCCTCTA

                     640       650       660       670       680       690       700
            ....|....|....|....|....|....|....|....|....|....|....|....|....|....|
KACC42870   CGTGGAAGCATATCTGCTTCAGGAGACCTAGCTCCACTCTCTTACATCGCTGGAACTCTCAGTGGAAACC
ASI4208     CGTGGAAGCATATCTGCTTCAGGAGACCTAGCTCCACTCTCTTACATCGCTGGAACTCTCAGTGGAAACC
ASI4049     CGTGGAAGCATATCTGCTTCAGGAGACCTAGCTCCACTCTCTTACATCGCTGGAACTCTCAGTGGAAACC
ASI4057     CGTGGAAGCATATCTGCTTCAGGAGACCTAGCTCCACTCTCTTACATCGCTGGAACTCTCAGTGGAAACC
ASI4166     CGTGGAAGCATATCTGCTTCAGGAGACCTAGCTCCACTCTCTTACATCGCTGGAACTCTCAGTGGAAACC
ASI4167     CGTGGAAGCATATCTGCTTCAGGAGACCTAGCTCCACTCTCTTACATCGCTGGAACTCTCAGTGGAAACC
ASI4169     CGTGGAAGCATATCTGCTTCAGGAGACCTAGCTCCACTCTCTTACATCGCTGGAACTCTCAGTGGAAACC

                     710       720       730       740       750       760       770
            ....|....|....|....|....|....|....|....|....|....|....|....|....|....|
KACC42870   CATCAATACGCGTCTTTGACGGCCCACGAAAATTTGGCTCCCGTAATATCGTGTCCTCGGTCGAAGCCCT
ASI4208     CATCAATACGCGTCTTTGACGGCCCACGAAAATTTGGCTCCCGTAATATCGTGTCCTCGGTCGAAGCCCT
ASI4049     CATCAATACGCGTCTTTGACGGCCCACGAAAATTTGGCTCCCGTAATATCGTGTCCTCGGTCGAAGCCCT
ASI4057     CATCAATACGCGTCTTTGACGGCCCACGAAAATTTGGCTCCCGTAATATCGTGTCCTCGGTCGAAGCCCT
ASI4166     CATCAATACGCGTCTTTGACGGCCCACGAAAATTTGGCTCCCGTAATATCGTGTCCTCGGTCGAAGCCCT
ASI4167     CATCAATACGCGTCTTTGACGGCCCACGAAAATTTGGCTCCCGTAATATCGTGTCCTCGGTCGAAGCCCT
ASI4169     CATCAATACGCGTCTTTGACGGCCCACGAAAATTTGGCTCCCGTAATATCGTGTCCTCGGTCGAAGCCCT

                     780       790       800       810       820       830       840
            ....|....|....|....|....|....|....|....|....|....|....|....|....|....|
KACC42870   CAAATCGCACAACATCGAGCCCCTCCCCCTTGCCTCCAAGGAACCTCTCGGGATTCTGAATGGAACAGCC
ASI4208     CAAATCGCACAACATCGAGCCCCTCCCCCTTGCCTCCAAGGAACCTCTCGGGATTCTGAATGGAACAGCC
ASI4049     CAAATCGCACAACATCGAGCCCCTCCCCCTTGCCTCCAAGGAACCTCTCGGGATTCTGAATGGAACAGCC
ASI4057     CAAATCGCACAACATCGAGCCCCTCCCCCTTGCCTCCAAGGAACCTCTCGGGATTCTGAATGGAACAGCC
ASI4166     CAAATCGCACAACATCGAGCCCCTCCCCCTTGCCTCCAAGGAACCTCTCGGGATTCTGAATGGAACAGCC
ASI4167     CAAATCGCACAACATCGAGCCCCTCCCCCTTGCCTCCAAGGAACCTCTCGGGATTCTGAATGGAACAGCC
ASI4169     CAAATCGCACAACATCGAGCCCCTCCCCCTTGCCTCCAAGGAACCTCTCGGGATTCTGAATGGAACAGCC

                     850       860       870       880       890       900       910
            ....|....|....|....|....|....|....|....|....|....|....|....|....|....|
KACC42870   TTTTCAGCCGCAGTCGCTTCGCTTGCTTTGAACGATGCCGTACATTTGACTTTACTTTCGCAAGTTTGTA
ASI4208     TTTTCAGCCGCAGTCGCTTCGCTTGCTTTGAACGATGCCGTACATTTGACTTTACTTTCTCAAGTTTGTA
ASI4049     TTTTCAGCCGCAGTCGCTTCGCTTGCTTTGAACGATGCCGTACATTTGACTTTACTTTCTCAAGTTTGTA
ASI4057     TTTTCAGCCGCAGTCGCTTCGCTTGCTTTGAACGATGCCGTACATTTGACTTTACTTTCTCAAGTTTGTA
ASI4166     TTTTCAGCCGCAGTCGCTTCGCTTGCTTTGAACGATGCCGTACATTTGACTTTACTTTCTCAAGTTTGTA
ASI4167     TTTTCAGCCGCAGTCGCTTCGCTTGCTTTGAACGATGCCGTACATTTGACTTTACTTTCTCAAGTTTGTA
ASI4169     TTTTCAGCCGCAGTCGCTTCGCTTGCTTTGAACGATGCCGTACATTTGACTTTACTTTCTCAAGTTTGTA

                     920       930       940       950       960       970       980
            ....|....|....|....|....|....|....|....|....|....|....|....|....|....|
KACC42870   CGGCGATGGGCGTAGAAGTACTGCTGGGAACCCGCGCATCGTTTGATCCATTCATTCATGCAGTCGCGAG
ASI4208     CGGCGATGGGCGTAGAAGTACTACTGGGAACCCGCGCATCGTTTGATCCATTCATTCATGCAGTCGCGAG
ASI4049     CGGCGATGGGCGTAGAAGTACTACTGGGAACCCGCGCATCGTTTGATCCATTCATTCATGCAGTCGCGAG
ASI4057     CGGCGATGGGCGTAGAAGTACTGCTGGGAACCCGCGCATCGTTTGATCCATTCATTCATGCAGTCGCGAG
ASI4166     CGGCGATGGGCGTAGAAGTACTACTGGGAACCCGCGCATCGTTTGATCCATTCATTCATGCAGTCGCGAG
ASI4167     CGGCGATGGGCGTAGAAGTACTACTGGGAACCCGCGCATCGTTTGATCCATTCATTCATGCAGTCGCGAG
ASI4169     CGGCGATGGGCGTAGAAGTACTACTGGGAACCCGCGCATCGTTTGATCCATTCATTCATGCAGTCGCGAG

                     990      1000      1010      1020      1030      1040      1050
            ....|....|....|....|....|....|....|....|....|....|....|....|....|....|
KACC42870   ACCACACCCAGGACAGATCGAGTGTGCGAAGATCATATGGGACTTGCTGGAAGACAGTCATTTTGCTCAA
ASI4208     ACCACACCCAGGACAGATCGAGTGTGCGAAGATCATATGGGACTTGCTGGAAGACAGTCATTTTGCTCAA
ASI4049     ACCACATCCAGGACAGATCGAGTGTGCGAAGATCATATGGGACTTGCTGGAAGACAGTCATTTTGCTCAA
ASI4057     ACCACATCCAGGACAGATCGAGTGTGCGAAGATCATATGGGACTTGCTGGAAGACAGTCATTTTGCTCAA
ASI4166     ACCACACCCAGGACAGATCGAGTGTGCGAAGATCATATGGGACTTGCTGGAAGACAGTCATTTTGCTCAA
ASI4167     ACCACACCCAGGACAGATCGAGTGTGCGAAGATCATATGGGACTTGCTGGAAGACAGTCATTTTGCTCAA
ASI4169     ACCACACCCAGGACAGATCGAGTGTGCGAAGATCATATGGGACTTGCTGGAAGACAGTCATTTTGCTCAA

                    1060      1070      1080      1090      1100      1110      1120
            ....|....|....|....|....|....|....|....|....|....|....|....|....|....|
KACC42870   CTTCACGAGGAAGAGGTGACGATCGCCGAGGATGAAGGCACCCTTAGACAGGATCGTTATGCCCTGCGTA
ASI4208     CTTCACGAGGAAGAGGTGACGATCGCCGAGGATGAAGGCACCCTTCGACAGGATCGTTATGCCCTGCGTA
ASI4049     CTTCACGAGGAAGAGGTGACGATCGCCGAGGATGAAGGCACCCTTCGACAGGATCGTTATGCCCTGCGTA
ASI4057     CTTCACGAGGAAGAGGTGACGATCGCCGAGGATGAAGGCACCCTTCGACAGGATCGTTATGCCCTGCGTA
ASI4166     CTTCACGAGGAAGAGGTGACGATCGCCGAGGATGAAGGCACCCTTCGACAGGATCGTTATGCCCTGCGTA
ASI4167     CTTCACGAGGAAGAGGTGACGATCGCCGAGGATGAAGGCACCCTTCGACAGGATCGTTATGCCCTGCGTA
ASI4169     CTTCACGAGGAAGAGGTGACGATCGCCGAGGATGAAGGCACCCTTCGACAGGATCGTTATGCCCTGCGTA
```

```
                    1130      1140      1150      1160      1170      1180      1190
           ....|....|....|....|....|....|....|....|....|....|....|....|....|....|
KACC42870  CTGCTCCCCAGTTTATCGGACCACAAATCGAGGACTTATTGGCATCCTTGGAAACCATAACGAGGGAGTG
ASI4208    CTGCTCCCCAGTTTATCGGACCACAAATCGAGGACTTATTGGCATCGTTGGAAACCATAACGAGGGAATG
ASI4049    CTGCTCCCCAGTTTATCGGACCACAAATCGAGGACTTATTGGCATCGTTGGAAACCATAACGAGGGAATG
ASI4057    CTGCTCCCCAGTTTATCGGACCACAAATCGAGGACTTATTGGCATCGTTGGAAACTATAACGAGGGAGTG
ASI4166    CTGCTCCCCAGTTTATCGGACCACAAATCGAGGACTTATTGGCATCGTTGGAAACCATAACGAGGGAATG
ASI4167    CTGCTCCCCAGTTTATCGGACCACAAATCGAGGACTTATTGGCATCGTTGGAAACCATAACGAGGGAATG
ASI4169    CTGCTCCCCAGTTTATCGGACCACAAATCGAGGACTTATTGGCATCGTTGGAAACCATAACGAGGGAATG

                    1200      1210      1220      1230      1240      1250      1260
           ....|....|....|....|....|....|....|....|....|....|....|....|....|....|
KACC42870  TAACTCAACTACCGATAACCCTCTTTCAGATGGGGAGACTGGCACAATTCACCATGGTGGCAACTTTCAA
ASI4208    TAACTCAACTACCGATAACCCTCTTTCAGATGGGGAGACTGGCACAATTCACCACGGTGGCAACTTTCAA
ASI4049    TAACTCAACTACCGATAACCCTCTTTCAGATGGGGAGACTGGCACAATTCACCATGGTGGCAACTTTCAA
ASI4057    TAACTCAACTACCGATAACCCTCTTTCAGATGGGGAGACTGGCACAATTCACCATGGTGGCAACTTTCAA
ASI4166    TAACTCAACTACCGATAACCCTCTTTCAGATGGGGAGACTGGCACAATTCACCACGGTGGCAACTTTCAA
ASI4167    TAACTCAACTACCGATAACCCTCTTTCAGATGGGGAGACTGGCACAATTCACCACGGTGGCAACTTTCAA
ASI4169    TAACTCAACTACCGATAACCCTCTTTCAGATGGGGAGACTGGCACAATTCACCACGGTGGCAACTTTCAA

                    1270      1280      1290      1300      1310      1320      1330
           ....|....|....|....|....|....|....|....|....|....|....|....|....|....|
KACC42870  GCGATGGCGGTAACAAACGCCATGGAGAAGACTCGTCTGAGCCTCCATCACATAGGCAAAATTATGTTTG
ASI4208    GCGATGGCGGTAACAAACGCCATGGAGAAGACTCGTCTGAGCCTCCATCACATAGGCAAAATTATGTTTG
ASI4049    GCGATGGCGGTAACAAACGCCATGGAGAAGACTCGTCTGAGCCTCCATCACATAGGCAAAATTATGTTTG
ASI4057    GCGATGGCGGTAACAAACGCCATGGAGAAGACTCGTCTGAGCCTCCATCACATAGGCAAAATTATGTTTG
ASI4166    GCGATGGCGGTAACAAACGCCATGGAGAAGACTCGTCTGAGCCTCCATCACATAGGCAAAATTATGTTTG
ASI4167    GCGATGGCGGTAACAAACGCCATGGAGAAGACTCGTCTGAGCCTCCATCACATAGGCAAAATTATGTTTG
ASI4169    GCGATGGCGGTAACAAACGCCATGGAGAAGACTCGTCTGAGCCTCCATCACATAGGCAAAATTATGTTTG

                    1340      1350      1360      1370      1380      1390      1400
           ....|....|....|....|....|....|....|....|....|....|....|....|....|....|
KACC42870  CTCAATGCGCTGAGCTAGTTGATCCTTCGATGAACCGTGGCCTGCCACCTTCTCTTGCGGCAACAGATCC
ASI4208    CTCAATGCGCTGAGCTAGTTGATCCTTCGATGAATCGTGGCCTGCCACCTTCTCTTGCGGCAACAGATCC
ASI4049    CTCAATGCGCTGAGCTAGTTGATCCTTCGATGAATCGTGGCCTGCCACCTTCTCTTGCGGCAACAGATCC
ASI4057    CTCAATGCGCTGAGCTAGTTGATCCTTCGATGAATCGTGGCCTGCCACCTTCTCTTGCGGCAACAGATCC
ASI4166    CTCAATGCGCTGAGCTAGTTGATCCTTCGATGAATCGTGGCCTGCCACCTTCTCTTGCGGCAACAGATCC
ASI4167    CTCAATGCGCTGAGCTAGTTGATCCTTCGATGAATCGTGGCCTGCCACCTTCTCTTGCGGCAACAGATCC
ASI4169    CTCAATGCGCTGAGCTAGTTGATCCTTCGATGAATCGTGGCCTGCCACCTTCTCTTGCGGCAACAGATCC

                    1410      1420      1430      1440      1450      1460      1470
           ....|....|....|....|....|....|....|....|....|....|....|....|....|....|
KACC42870  TTCGCTGAACTACCATGGCAAAGGCATTGACATTGCTGCGGCTGCATATGTATCAGAACTAGGTTATCTT
ASI4208    TTCCTTGAACTATCATGGCAAAGGCATTGACATTGCCGCGGCTGCATACGTATCAGAATTAGGTTATCTT
ASI4049    TTCCTTGAACTATCATGGCAAAGGCATTGACATTGCCGCGGCTGCATACGTATCAGAATTAGGTTATCTT
ASI4057    TTCGTTGAACTATCATGGCAAAGGCATTGACATTGCCGCGGCTGCATACGTATCAGAATTAGGTTATCTT
ASI4166    TTCCTTGAACTATCATGGCAAAGGCATTGACATTGCCGCGGCTGCATACGTATCAGAATTAGGTTATCTT
ASI4167    TTCCTTGAACTATCATGGCAAAGGCATTGACATTGCCGCGGCTGCATACGTATCAGAATTAGGTTATCTT
ASI4169    TTCCTTGAACTATCATGGCAAAGGCATTGACATTGCCGCGGCTGCATACGTATCAGAATTAGGTTATCTT

                    1480      1490      1500      1510      1520      1530      1540
           ....|....|....|....|....|....|....|....|....|....|....|....|....|....|
KACC42870  GCGAACCCTGTGTCTACGCATATTCAATCGGCGGAGATGCATAACCAAGCTGTCAACTCGTTGGCTCTTA
ASI4208    GCGAACCCTGTGTCTACGCATATTCAATCGGCGGAGATGCATAACCAAGCTGTCAACTCGTTGGCTCTTA
ASI4049    GCGAACCCTGTGTCTACGCATATTCAATCGGCGGAGATGCATAACCAAGCTGTCAACTCGTTGGCTCTTA
ASI4057    GCGAACCCTGTGTCTACGCATATTCAATCGGCGGAGATGCATAACCAAGCTGTCAACTCGTTGGCTCTTA
ASI4166    GCGAACCCTGTGTCTACGCATATTCAATCGGCGGAGATGCATAACCAAGCTGTCAACTCGTTGGCTCTTA
ASI4167    GCGAACCCTGTGTCTACGCATATTCAATCGGCGGAGATGCATAACCAAGCTGTCAACTCGTTGGCTCTTA
ASI4169    GCGAACCCTGTGTCTACGCATATTCAATCGGCGGAGATGCATAACCAAGCTGTCAACTCGTTGGCTCTTA

                    1550      1560      1570      1580      1590      1600      1610
           ....|....|....|....|....|....|....|....|....|....|....|....|....|....|
KACC42870  TTTCTGGTCGCGCAACGATCAATTCATTGGAGACGTTGTCCATTTTGATCTCCTCTTACCTTTACTTGAT
ASI4208    TCTCTGGTCGCGCGACGATCAACTCATTGGAGACGTTGTCCATTTTGATCTCTTCTTACCTTTACTTGAT
ASI4049    TTTCTGGTCGCGCGACGATCAACTCATTGGAGACGTTGTCCATTTTGATCTCTTCTTACCTTTACTTGAT
ASI4057    TTTCTGGTCGCGCGACGATCAACTCATTGGAGACGTTGTCCATTTTGATCTCTTCTTACCTTTACTTGAT
ASI4166    TCTCTGGTCGCGCGACGATCAACTCATTGGAGACGTTGTCCATTTTGATCTCTTCTTACCTTTACTTGAT
ASI4167    TCTCTGGTCGCGCGACGATCAACTCATTGGAGACGTTGTCCATTTTGATCTCTTCTTACCTTTACTTGAT
ASI4169    TCTCTGGTCGCGCGACGATCAACTCATTGGAGACGTTGTCCATTTTGATCTCTTCTTACCTTTACTTGAT

                    1620      1630      1640      1650      1660      1670      1680
           ....|....|....|....|....|....|....|....|....|....|....|....|....|....|
KACC42870  CTGCCAAGCCTATGACCTCCGTGCGCTTCAAGAAGAATTTTCAGACGGGGCAACTACTATCGTGAAAAGG
ASI4208    CTGCCAAGCCTATGACCTCCGTGCGCTCCAAGAAGAGTTTTCAGATGGGGCAACTACTATCGTGAAAAGG
ASI4049    CTGCCAAGCCTATGACCTCCGTGCGCTCCAAGAAGAGTTTTCAGATGGGGCAACTACTATCGTGAAAAGG
ASI4057    CTGCCAAGCCTATGACCTCCGTGCGCTCCAAGAAGAGTTTTCAGATGGGGCAACTACTATCGTGAAAAGG
ASI4166    CTGCCAAGCCTATGACCTCCGTGCGCTCCAAGAAGAGTTTTCAGATGGGGCAACTACTATCGTGAAAAGG
ASI4167    CTGCCAAGCCTATGACCTCCGTGCGCTCCAAGAAGAGTTTTCAGATGGGGCAACTACTATCGTGAAAAGG
ASI4169    CTGCCAAGCCTATGACCTCCGTGCGCTCCAAGAAGAGTTTTCAGATGGGGCAACTACTATCGTGAAAAGG
```

```
                  1690      1700      1710      1720      1730      1740      1750
             ....|....|....|....|....|....|....|....|....|....|....|....|....|....|
KACC42870    GAGATCGCAGAGCGTTTCTCAGACTGCCTTTCGTTCTCGGACCTCTCATCACTCACCAACAAGGTGGTGA
ASI4208      GAGATCGCAGAGCGTTTCTCAGACTGCCTTTCGTTCTCCGACCTCTCATCACTCACCAACAAGGTGGTGA
ASI4049      GAGATCGCAGAGCGTTTCTCAGACTGCCTTTCGTTCTCCGACCTCTCATCACTCACCAACAAGGTGGTGA
ASI4057      GAGATCGCAGAGCGTTTCTCAGACTGCCTTTCGTTCTCCGACCTCTCATCACTCACCAACAAGGTGGTGA
ASI4166      GAGATCGCAGAGCGTTTCTCAGACTGCCTTTCGTTCTCCGACCTCTCATCACTCACCAACAAGGTGGTGA
ASI4167      GAGATCGCAGAGCGTTTCTCAGACTGCCTTTCGTTCTCCGACCTCTCATCACTCACCAACAAGGTGGTGA
ASI4169      GAGATCGCAGAGCGTTTCTCAGACTGCCTTTCGTTCTCCGACCTCTCATCACTCACCAACAAGGTGGTGA

                  1760      1770      1780      1790      1800      1810      1820
             ....|....|....|....|....|....|....|....|....|....|....|....|....|....|
KACC42870    AAGTCGTCTTGGAATCTCTAGATGAAACGACTACCATGGACGCTTCAGAAAGGATGGTGAAAGTCGCTGC
ASI4208      AAGTCGTCTTGGAATCTCTAGATGAAACGACTACCATGGACGCTTCAGAAAGGATGGTGAAAGTCGCTGC
ASI4049      AAGTCGTCTTGGAATCTCTAGATGAAACGACTACCATGGACGCTTCAGAAAGGATGGTGAAAGTCGCTGC
ASI4057      AAGTCGTCTTGGAATCTCTAGATGAAACGACTACCATGGACGCTTCAGAAAGGATGGTGAAAGTCGCTGC
ASI4166      AAGTCGTCTTGGAATCTCTAGATGAAACGACTACCATGGACGCTTCAGAAAGGATGGTGAAAGTCGCTGC
ASI4167      AAGTCGTCTTGGAATCTCTAGATGAAACGACTACCATGGACGCTTCAGAAAGGATGGTGAAAGTCGCTGC
ASI4169      AAGTCGTCTTGGAATCTCTAGATGAAACGACTACCATGGACGCTTCAGAAAGGATGGTGAAAGTCGCTGC

                  1830      1840      1850      1860      1870      1880      1890
             ....|....|....|....|....|....|....|....|....|....|....|....|....|....|
KACC42870    ATCCACGTCCACGGTCCTCGTCGACTTTTTATCCGAGTGCTCGTTCACCAAGGACACCACCGACGTTGGC
ASI4208      ATCCACGTCCACGGTCCTCGTCGACTTTTTATCCGAGTGCTCGTTCACCAAGGACACCACCGACATTGGC
ASI4049      ATCCACGTCCACGGTCCTCGTCGACTTTTTATCCGAGTGCTCGTTCACCAAGGACACCACCGACATTGGC
ASI4057      ATCCACGTCCACGGTCCTCGTCGACTTTTTATCCGAGTGCTCGTTCACCAAGGACACCACCGACATTGGC
ASI4166      ATCCACGTCCACGGTCCTCGTCGACTTTTTATCCGAGTGCTCGTTCACCAAGGACACCACCGACATTGGC
ASI4167      ATCCACGTCCACGGTCCTCGTCGACTTTTTATCCGAGTGCTCGTTCACCAAGGACACCACCGACATTGGC
ASI4169      ATCCACGTCCACGGTCCTCGTCGACTTTTTATCCGAGTGCTCGTTCACCAAGGACACCACCGACATTGGC

                  1900      1910      1920      1930      1940      1950      1960
             ....|....|....|....|....|....|....|....|....|....|....|....|....|....|
KACC42870    TCTGCTCTCTCCTATATCCCGACCTTCCGTTCGCGTGTGGCATCTAACATGACCATCCTGCTCGATCGGT
ASI4208      TCTGCTCTCTCCTATATCCCGACCTTCCGTTCGCGTGTGGCATCTAACATGACCATCCTGCTCGATCGGT
ASI4049      TCTGCTCTCTCCTATATCCCGACCTTCCGTTCGCGTGTGGCATCTAACATGACCATCCTGCTCGATCGGT
ASI4057      TCTGCTCTCTCCTATATCCCGACCTTCCGTTCGCGTGTGGCATCTAACATGACCATCCTGCTCGATCGGT
ASI4166      TCTGCTCTCTCCTATATCCCGACCTTCCGTTCGCGTGTGGCATCTAACATGACCATCCTGCTCGATCGGT
ASI4167      TCTGCTCTCTCCTATATCCCGACCTTCCGTTCGCGTGTGGCATCTAACATGACCATCCTGCTCGATCGGT
ASI4169      TCTGCTCTCTCCTATATCCCGACCTTCCGTTCGCGTGTGGCATCTAACATGACCATCCTGCTCGATCGGT

                  1970      1980      1990      2000      2010      2020      2030
             ....|....|....|....|....|....|....|....|....|....|....|....|....|....|
KACC42870    TACGGAAGGCATACCTTAGTGGTGAACGCGGGCCGGCGCCTGCCAGCAAGCTCTTGAAGAAAACCAAGCC
ASI4208      TACGGAAGGCATACCTTAGTGGTGAACGCGGGCCGGCGCCTGCCAGCAAGCTCTTGAAGAAAACCAAGCC
ASI4049      TACGGAAGGCATACCTTAGTGGTGAACGCGGGCCGGCGCCTGCCAGCAAGCTCTTGAAGAAAACCAAGCC
ASI4057      TACGGAAGGCATACCTTAGTGGTGAACGCGGGCCGGCGCCTGCCAGCAAGCTCTTGAAGAAAACCAAGCC
ASI4166      TACGGAAGGCATACCTTAGTGGTGAACGCGGGCCGGCGCCTGCCAGCAAGCTCTTGAAGAAAACCAAGCC
ASI4167      TACGGAAGGCATACCTTAGTGGTGAACGCGGGCCGGCGCCTGCCAGCAAGCTCTTGAAGAAAACCAAGCC
ASI4169      TACGGAAGGCATACCTTAGTGGTGAACGCGGGCCGGCGCCTGCCAGCAAGCTCTTGAAGAAAACCAAGCC

                  2040      2050      2060      2070      2080      2090      2100
             ....|....|....|....|....|....|....|....|....|....|....|....|....|....|
KACC42870    ACTGTATGAATTTGTTCGTTTGACTTTGGGCATCCGCATGCATGGTTCCGAAAACTATAACCTGTTCGCC
ASI4208      ACTGTATGAATTTGTTCGTTTGACTTTGGGCATCCGCATGCATGGTTCCGAAAACTATAACCTGTTCGCC
ASI4049      ACTGTATGAATTTGTTCGTTTGACTTTGGGCATCCGCATGCATGGTTCCGAAAACTATAACCTGTTCGCC
ASI4057      ACTGTATGAATTTGTTCGTTTGACTTTGGGCATCCGCATGCATGGTTCCGAAAACTATAACCTGTTCGCC
ASI4166      ACTGTATGAATTTGTTCGTTTGACTTTGGGCATCCGCATGCATGGTTCCGAAAACTATAACCTGTTCGCC
ASI4167      ACTGTATGAATTTGTTCGTTTGACTTTGGGCATCCGCATGCATGGTTCCGAAAACTATAACCTGTTCGCC
ASI4169      ACTGTATGAATTTGTTCGTTTGACTTTGGGCATCCGCATGCATGGTTCCGAAAACTATAACCTGTTCGCC

                  2110      2120      2130      2140      2150      2160      2170
             ....|....|....|....|....|....|....|....|....|....|....|....|....|....|
KACC42870    AATGGGCATGGCTTCGACGAGGTCACGACTGGTCAAAGTGTATCCCTTATCCACGAGGCTATCCGTGATG
ASI4208      AATGGGCATGGCTTCGACGAGGTCACGACTGGTCAAAGTGTATCCCTTATCCACGAGGCTATCCGTGATG
ASI4049      AATGGGCATGGCTTCGACGAGGTCACGACTGGTCAAAGTGTATCCCTTATCCACGAGGCTATCCGTGATG
ASI4057      AATGGGCATGGCTTCGACGAGGTCACGACTGGTCAAAGTGTATCCCTTATCCACGAGGCTATCCGTGATG
ASI4166      AATGGGCATGGCTTCGACGAGGTCACGACTGGTCAAAGTGTATCCCTTATCCACGAGGCTATCCGTGATG
ASI4167      AATGGGCATGGCTTCGACGAGGTCACGACTGGTCAAAGTGTATCCCTTATCCACGAGGCTATCCGTGATG
ASI4169      AATGGGCATGGCTTCGACGAGGTCACGACTGGTCAAAGTGTATCCCTTATCCACGAGGCTATCCGTGATG

                  2180      2190      2200
             ....|....|....|....|....|....|....|...
KACC42870    GAAAACTACAAGCCGCTGTTGTCAGCATGCTTGCTTGA
ASI4208      GAAAACTACAAGCCGCTGTTGTCAGCATGCTTGCTTGA
ASI4049      GAAAACTACAAGCCGCTGTTGTCAGCATGCTTGCTTGA
ASI4057      GAAAACTACAAGCCGCTGTTGTCAGCATGCTTGCTTGA
ASI4166      GAAAACTACAAGCCGCTGTTGTCAGCATGCTTGCTTGA
ASI4167      GAAAACTACAAGCCGCTGTTGTCAGCATGCTTGCTTGA
ASI4169      GAAAACTACAAGCCGCTGTTGTCAGCATGCTTGCTTGA
```

(a)

```
                    10         20         30         40         50         60         70
KACC42870  MAILSETRTVRRTSSAPAPAASLLERKDPASYTTHSAKLLEKFVASYRELESYKHGKTVHVDGQTLSIAA
ASI4208    MAILSETRTVRRTSSAPAPAASLLERKDPASYTTHSAKLLEKFVASYRELESYKHGKTVHVDGQTLSIAA
ASI4049    MAILSETRTVRRTSSAPAPAASLLERKDPASYTTHSAKLLEKFVASYRELESYKHGKTVHVDGQTLSIAA
ASI4057    MAILSETRTVRRTSSAPAPAASLLERKDPASYTTHSAKLLEKFVASYRELESYKHGKTVHVDGQTLSIAA
ASI4166    MAILSETRTVRRTSSAPAPAASLLERKDPASYTTHSAKLLEKFVASYRELESYKHGKTVHVDGQTLSIAA
ASI4167    MAILSETRTVRRTSSAPAPAASLLERKDPASYTTHSAKLLEKFVASYRELESYKHGKTVHVDGQTLSIAA
ASI4169    MAILSETRTVRRTSSAPAPAASLLERKDPASYTTHSAKLLEKFVASYRELESYKHGKTVHVDGQTLSIAA

                    80         90        100        110        120        130        140
KACC42870  VTAAARYDTPVHLTSSQDVKKRVAKSRLIIADKVDNGISVYGVSTGFGGSADTRTDLPIVLGHSLLQMQH
ASI4208    VTAAARYDTPVHLTSSQDVKKRVAKSRLIIADKVDNGISVYGVSTGFGGSADTRTDLPIVLGHSLLQMQH
ASI4049    VTAAARYDTPVHLTSSQDVKKRVAKSRLIIADKVDNGISVYGVSTGFGGSADTRTDLPIVLGHSLLQMQH
ASI4057    VTAAARYDTPVHLTSSQDVKKRVAKSRLIIADKVDNGISVYGVSTGFGGSADTRTDLPIVLGHSLLQMQH
ASI4166    VTAAARYDTPVHLTSSQDVKKRVAKSRLIIADKVDNGISVYGVSTGFGGSADTRTDLPIVLGHSLLQMQH
ASI4167    VTAAARYDTPVHLTSSQDVKKRVAKSRLIIADKVDNGISVYGVSTGFGGSADTRTDLPIVLGHSLLQMQH
ASI4169    VTAAARYDTPVHLTSSQDVKKRVAKSRLIIADKVDNGISVYGVSTGFGGSADTRTDLPIVLGHSLLQMQH

                   150        160        170        180        190        200        210
KACC42870  SGVLPSSTQALEALPLLDPMGSTVMPESWVRGAILIRMNSLIRGHSGVRWELIEKMKALLDANITPIVPL
ASI4208    SGVLPSSTQALEALPLLDPMGSTVMPESWVRGAILIRMNSLIRGHSGVRWELIEKMKALLDANITPIVPL
ASI4049    SGVLPSSTQALEALPLLDPMGSTVMPESWVRGAILIRMNSLIRGHSGVRWELIEKMKALLDANITPIVPL
ASI4057    SGVLPSSTQALEALPLLDPMGSTVMPESWVRGAILIRMNSLIRGHSGVRWELIEKMKALLDANITPIVPL
ASI4166    SGVLPSSTQALEALPLLDPMGSTVMPESWVRGAILIRMNSLIRGHSGVRWELIEKMKALLDANITPIVPL
ASI4167    SGVLPSSTQALEALPLLDPMGSTVMPESWVRGAILIRMNSLIRGHSGVRWELIEKMKALLDANITPIVPL
ASI4169    SGVLPSSTQALEALPLLDPMGSTVMPESWVRGAILIRMNSLIRGHSGVRWELIEKMKALLDANITPIVPL

                   220        230        240        250        260        270        280
KACC42870  RGSISASGDLAPLSYIAGTLSGNPSIRVFDGPRKFGSRNIVSSVEALKSHNIEPLPLASKEPLGILNGTA
ASI4208    RGSISASGDLAPLSYIAGTLSGNPSIRVFDGPRKFGSRNIVSSVEALKSHNIEPLPLASKEPLGILNGTA
ASI4049    RGSISASGDLAPLSYIAGTLSGNPSIRVFDGPRKFGSRNIVSSVEALKSHNIEPLPLASKEPLGILNGTA
ASI4057    RGSISASGDLAPLSYIAGTLSGNPSIRVFDGPRKFGSRNIVSSVEALKSHNIEPLPLASKEPLGILNGTA
ASI4166    RGSISASGDLAPLSYIAGTLSGNPSIRVFDGPRKFGSRNIVSSVEALKSHNIEPLPLASKEPLGILNGTA
ASI4167    RGSISASGDLAPLSYIAGTLSGNPSIRVFDGPRKFGSRNIVSSVEALKSHNIEPLPLASKEPLGILNGTA
ASI4169    RGSISASGDLAPLSYIAGTLSGNPSIRVFDGPRKFGSRNIVSSVEALKSHNIEPLPLASKEPLGILNGTA

                   290        300        310        320        330        340        350
KACC42870  FSAAVASLALNDAVHLTLLSQVCTAMGVEVLLGTRASFDPFIHAVARPHPGQIECAKIIWDLLEDSHFAQ
ASI4208    FSAAVASLALNDAVHLTLLSQVCTAMGVEVLLGTRASFDPFIHAVARPHPGQIECAKIIWDLLEDSHFAQ
ASI4049    FSAAVASLALNDAVHLTLLSQVCTAMGVEVLLGTRASFDPFIHAVARPHPGQIECAKIIWDLLEDSHFAQ
ASI4057    FSAAVASLALNDAVHLTLLSQVCTAMGVEVLLGTRASFDPFIHAVARPHPGQIECAKIIWDLLEDSHFAQ
ASI4166    FSAAVASLALNDAVHLTLLSQVCTAMGVEVLLGTRASFDPFIHAVARPHPGQIECAKIIWDLLEDSHFAQ
ASI4167    FSAAVASLALNDAVHLTLLSQVCTAMGVEVLLGTRASFDPFIHAVARPHPGQIECAKIIWDLLEDSHFAQ
ASI4169    FSAAVASLALNDAVHLTLLSQVCTAMGVEVLLGTRASFDPFIHAVARPHPGQIECAKIIWDLLEDSHFAQ

                   360        370        380        390        400        410        420
KACC42870  LHEEEVTIAEDEGTLRQDRYALRTAPQFIGPQIEDLLASLETITRECNSTTDNPLSDGETGTIHHGGNFQ
ASI4208    LHEEEVTIAEDEGTLRQDRYALRTAPQFIGPQIEDLLASLETITRECNSTTDNPLSDGETGTIHHGGNFQ
ASI4049    LHEEEVTIAEDEGTLRQDRYALRTAPQFIGPQIEDLLASLETITRECNSTTDNPLSDGETGTIHHGGNFQ
ASI4057    LHEEEVTIAEDEGTLRQDRYALRTAPQFIGPQIEDLLASLETITRECNSTTDNPLSDGETGTIHHGGNFQ
ASI4166    LHEEEVTIAEDEGTLRQDRYALRTAPQFIGPQIEDLLASLETITRECNSTTDNPLSDGETGTIHHGGNFQ
ASI4167    LHEEEVTIAEDEGTLRQDRYALRTAPQFIGPQIEDLLASLETITRECNSTTDNPLSDGETGTIHHGGNFQ
ASI4169    LHEEEVTIAEDEGTLRQDRYALRTAPQFIGPQIEDLLASLETITRECNSTTDNPLSDGETGTIHHGGNFQ

                   430        440        450        460        470        480        490
KACC42870  AMAVTNAMEKTRLSLHHIGKIMFAQCAELVDPSMNRGLPPSLAATDPSLNYHGKGIDIAAAAYVSELGYL
ASI4208    AMAVTNAMEKTRLSLHHIGKIMFAQCAELVDPSMNRGLPPSLAATDPSLNYHGKGIDIAAAAYVSELGYL
ASI4049    AMAVTNAMEKTRLSLHHIGKIMFAQCAELVDPSMNRGLPPSLAATDPSLNYHGKGIDIAAAAYVSELGYL
ASI4057    AMAVTNAMEKTRLSLHHIGKIMFAQCAELVDPSMNRGLPPSLAATDPSLNYHGKGIDIAAAAYVSELGYL
ASI4166    AMAVTNAMEKTRLSLHHIGKIMFAQCAELVDPSMNRGLPPSLAATDPSLNYHGKGIDIAAAAYVSELGYL
ASI4167    AMAVTNAMEKTRLSLHHIGKIMFAQCAELVDPSMNRGLPPSLAATDPSLNYHGKGIDIAAAAYVSELGYL
ASI4169    AMAVTNAMEKTRLSLHHIGKIMFAQCAELVDPSMNRGLPPSLAATDPSLNYHGKGIDIAAAAYVSELGYL

                   500        510        520        530        540        550        560
KACC42870  ANPVSTHIQSAEMHNQAVNSLALISGRATINSLETLSILISSYLYLICQAYDLRALQEEFSDGATTIVKR
ASI4208    ANPVSTHIQSAEMHNQAVNSLALISGRATINSLETLSILISSYLYLICQAYDLRALQEEFSDGATTIVKR
ASI4049    ANPVSTHIQSAEMHNQAVNSLALISGRATINSLETLSILISSYLYLICQAYDLRALQEEFSDGATTIVKR
ASI4057    ANPVSTHIQSAEMHNQAVNSLALISGRATINSLETLSILISSYLYLICQAYDLRALQEEFSDGATTIVKR
ASI4166    ANPVSTHIQSAEMHNQAVNSLALISGRATINSLETLSILISSYLYLICQAYDLRALQEEFSDGATTIVKR
ASI4167    ANPVSTHIQSAEMHNQAVNSLALISGRATINSLETLSILISSYLYLICQAYDLRALQEEFSDGATTIVKR
ASI4169    ANPVSTHIQSAEMHNQAVNSLALISGRATINSLETLSILISSYLYLICQAYDLRALQEEFSDGATTIVKR
```

```
                570       580       590       600       610       620       630
KACC42870  EIAERFSDCLSFSDLSSLTNKVVKVVLESLDETTTMDASERMVKVAASTSTVLVDFLSECSFTKDTTDVG
ASI4208    EIAERFSDCLSFSDLSSLTNKVVKVVLESLDETTTMDASERMVKVAASTSTVLVDFLSECSFTKDTTDIG
ASI4049    EIAERFSDCLSFSDLSSLTNKVVKVVLESLDETTTMDASERMVKVAASTSTVLVDFLSECSFTKDTTDIG
ASI4057    EIAERFSDCLSFSDLSSLTNKVVKVVLESLDETTTMDASERMVKVAASTSTVLVDFLSECSFTKDTTDIG
ASI4166    EIAERFSDCLSFSDLSSLTNKVVKVVLESLDETTTMDASERMVKVAASTSTVLVDFLSECSFTKDTTDIG
ASI4167    EIAERFSDCLSFSDLSSLTNKVVKVVLESLDETTTMDASERMVKVAASTSTVLVDFLSECSFTKDTTDIG
ASI4169    EIAERFSDCLSFSDLSSLTNKVVKVVLESLDETTTMDASERMVKVAASTSTVLVDFLSECSFTKDTTDIG

                640       650       660       670       680       690       700
KACC42870  SALSYIPTFRSRVASNMTILLDRLRKAYLSGERGPAPASKLLKKTKPLYEFVRLTLGIRMHGSENYNLFA
ASI4208    SALSYIPTFRSRVASNMTILLDRLRKAYLSGERGPAPASKLLKKTKPLYEFVRLTLGIRMHGSENYNLFA
ASI4049    SALSYIPTFRSRVASNMTILLDRLRKAYLSGERGPAPASKLLKKTKPLYEFVRLTLGIRMHGSENYNLFA
ASI4057    SALSYIPTFRSRVASNMTILLDRLRKAYLSGERGPAPASKLLKKTKPLYEFVRLTLGIRMHGSENYNLFA
ASI4166    SALSYIPTFRSRVASNMTILLDRLRKAYLSGERGPAPASKLLKKTKPLYEFVRLTLGIRMHGSENYNLFA
ASI4167    SALSYIPTFRSRVASNMTILLDRLRKAYLSGERGPAPASKLLKKTKPLYEFVRLTLGIRMHGSENYNLFA
ASI4169    SALSYIPTFRSRVASNMTILLDRLRKAYLSGERGPAPASKLLKKTKPLYEFVRLTLGIRMHGSENYNLFA

                710       720       730
KACC42870  NGHGFDEVTTGQSVSLIHEAIRDGKLQAAVVSMLA*
ASI4208    NGHGFDEVTTGQSVSLIHEAIRDGKLQAAVVSMLA*
ASI4049    NGHGFDEVTTGQSVSLIHEAIRDGKLQAAVVSMLA*
ASI4057    NGHGFDEVTTGQSVSLIHEAIRDGKLQAAVVSMLA*
ASI4166    NGHGFDEVTTGQSVSLIHEAIRDGKLQAAVVSMLA*
ASI4167    NGHGFDEVTTGQSVSLIHEAIRDGKLQAAVVSMLA*
ASI4169    NGHGFDEVTTGQSVSLIHEAIRDGKLQAAVVSMLA*
```

(b)

Figure S2. Alignments of phenylalanine ammonia-lyase 2 (*Fvpal2*) genes of *Flammulina velutipes* strains. (**a**) cDNA sequences; (**b**) amino acid sequences. *F. velutipes* KACC42870 (non-white strain), *F. velutipes* ASI4208 (non-white strain), *F. velutipes* ASI4049 (non-white strain), *F. velutipes* ASI4057 (non-white strain), *F. velutipes* ASI4166 (white strain), *F. velutipes* ASI4167 (white strain), *F. velutipes* ASI4169 (white strain).
