# Supplementary material for: Phenylalanine Ammonia-Lyase: A Key Gene for Color Discrimination of Edible Mushroom Flammulina velutipes
Source: J Fungi (Basel). 2023 Mar 9;9(3):339. doi: 10.3390/jof9030339 (PMC10053379; doi:10.3390/jof9030339)
Supplement: Supplementary file 1 [file jof-09-00339-s001.zip › Figure S1.pdf]

```
                    10        20        30        40        50        60        70
KACC42870  ATGCCTTCAGAACTCTTCGACCTCGACAATGCCAGGGCTGCCCGCGACACTTTCCTAGACGCGCGCCGTA
ASI4208    ATGCCTTCAGAACTCTTCGACCTCGACAATGCCAGGGCTGCCCGCGACACTTTCCTAGACGCGCGCCGTA
ASI4049    ATGCCTTCAGAACTGTTTGACCTCGACAATGCCAGGGCTGCCCGCGACACTTTCCTAGACGCGCGCCGTA
ASI4057    ATGCCTTCAGAACTGTTTGACCTCGACAATGCCAGGGCTGCCCGCGACACTTTCCTAGACGCGCGCCGTA
ASI4166    ATGCCTTCAGAACTCTTCGACCTCGACAATGCCAGGGCTGCCCGCGACACTTTCCTAGACGCGCGCCGTA
ASI4167    ATGCCTTCAGAACTCTTCGACCTCGACAATGCCAGGGCTGCCCGCGACACTTTCCTAGACGCGCGCCGTA
ASI4169    ATGCCTTCAGAACTCTTCGACCTCGACAATGCCAGGGCTGCCCGCGACACTTTCCTAGACGCGCGCCGTA

                    80        90       100       110       120       130       140
KACC42870  CTGCTACCCTATTGCACAAATTTCTTGATAGCCACCGGGAACTCAAGAGCTACAAGAATGGACGCACCAT
ASI4208    CTGCTACCCTATTGCACAAATTTCTTGATAGCCACCGGGAACTCAAGAGCTACAAGAATGGACGCACCAT
ASI4049    CTGCTACCCTATTGCACAAATTTCTTGATAGCCACCGGGAGCTCAAGAGCTACAAGAATGGACGCACCAT
ASI4057    CTGCTACCCTATTGCACAAATTTCTTGATAGCCACCGGGAGCTCAAGAGCTACAAGAATGGACGCACCAT
ASI4166    CTGCTACCCTATTGCACAAATTTCTTGATAGCCACCGGGAACTCAAGAGCTACAAGAATGGACGCACCAT
ASI4167    CTGCTACCCTATTGCACAAATTTCTTGATAGCCACCGGGAACTCAAGAGCTACAAGAATGGACGCACCAT
ASI4169    CTGCTACCCTATTGCACAAATTTCTTGATAGCCACCGGGAACTCAAGAGCTACAAGAATGGACGCACCAT

                   150       160       170       180       190       200       210
KACC42870  TAATGTAGATGGCCATACACTGTCTCTCGCCGCTGTGACCGCCGCTGCGCGCTACAACGCCAACGTTGAG
ASI4208    TAATGTAGATGGCCATACACTGTCTCTCGCCGCTGTGACCGCCGCTGCGCGCTACAACGCCAACGTTGAG
ASI4049    TAATGTAGATGGCCATACACTGTCTCTCGCCGCTGTGACCGCCGCTGCGCGCTACAACGCCAACGTTGAG
ASI4057    TAATGTAGATGGCCATACACTGTCTCTCGCCGCTGTGACCGCCGCTGCGCGCTACAACGCCAACGTTGAG
ASI4166    TAATGTAGATGGCCATACACTGTCTCTCGCCGCTGTGACCGCCGCTGCGCGCTACAACGCCAACGTTGAG
ASI4167    TAATGTAGATGGCCATACACTGTCTCTCGCCGCTGTGACCGCCGCTGCGCGCTACAACGCCAACGTTGAG
ASI4169    TAATGTAGATGGCCATACACTGTCTCTCGCCGCTGTGACCGCCGCTGCGCGCTACAACGCCAACGTTGAG

                   220       230       240       250       260       270       280
KACC42870  TTGAGTCAGAGTGCACAGGTCAAGGAAGGCGTCGAGAAGAGTCGCGCCGTCATCGCTGAAAAGGTGGAGC
ASI4208    TTGAGTCAGAGTGCACAGGTCAAGGAAGGCGTCGAGAAGAGTCGCGCCGTCATCGCTGAAAAGGTGGAGC
ASI4049    TTGAGTCAGAGTGCACAGGTCAAGGAAGGCGTCGAGAAGAGTCGCGCCGTCATCGCTGAAAAGGTGGAGC
ASI4057    TTGAGTCAGAGTGCACAGGTCAAGGAAGGCGTCGAGAAGAGTCGCGCCGTCATCGCTGAAAAGGTGGAGC
ASI4166    TTGAGTCAGAGTGCACAGGTCAAGGAAGGCGTCGAGAAGAGTCGCGCCGTCATCGCTGAAAAGGTGGAGC
ASI4167    TTGAGTCAGAGTGCACAGGTCAAGGAAGGCGTCGAGAAGAGTCGCGCCGTCATCGCTGAAAAGGTGGAGC
ASI4169    TTGAGTCAGAGTGCACAGGTCAAGGAAGGCGTCGAGAAGAGTCGCGCCGTCATCGCTGAAAAGGTGGAGC

                   290       300       310       320       330       340       350
KACC42870  AAGGCACTAGTGTCTACGGCGTCTCCACTGGCTTTGGTGGCAGCGCGGACACGCGCACTGACCAACCTCT
ASI4208    AAGGCACTAGTGTCTACGGCGTCTCCACTGGCTTTGGTGGCAGCGCGGACACGCGCACTGACCAACCTCT
ASI4049    AAGGCACTAGTGTCTACGGCGTCTCCACTGGCTTTGGTGGCAGCGCGGACACGCGCACTGACCAGCCTCT
ASI4057    AAGGCACTAGTGTCTACGGCGTCTCCACTGGCTTTGGTGGCAGCGCGGACACGCGCACTGACCAGCCTCT
ASI4166    AAGGCACTAGTGTCTACGGCGTCTCCACTGGCTTTGGTGGCAGCGCGGACAC------TGACCAACCTCT
ASI4167    AAGGCACTAGTGTCTACGGCGTCTCCACTGGCTTTGGTGGCAGCGCGGACAC------TGACCAACCTCT
ASI4169    AAGGCACTAGTGTCTACGGCGTCTCCACTGGCTTTGGTGGCAGCGCGGACAC------TGACCAACCTCT

                   360       370       380       390       400       410       420
KACC42870  CAAGCTGCAGCAGGCTCTTCTTCAACATCAGCATGCTGGCGTTCTCCCCAGCTCAAGCAAGACTCTAGGT
ASI4208    CAAGCTGCAGCAGGCTCTTCTTCAACATCAGCATGCTGGCGTTCTCCCCAGCTCAAGCAAGACTCTAGGT
ASI4049    CAAGCTGCAGCAGGCTCTTCTTCAACATCAGCATGCTGGCGTTCTCCCCAGCTCAAGCAAGACTCTAGGT
ASI4057    CAAGCTGCAGCAGGCTCTTCTTCAACATCAGCATGCTGGCGTTCTCCCCAGCTCAAGCAAGACTCTAGGT
ASI4166    CAAGCTGCAGCAGGCTCTTCTTCAACATCAGCATGCTGGCGTTCTCCCCAGCTCAAGCAAGACTCTAGGT
ASI4167    CAAGCTGCAGCAGGCTCTTCTTCAACATCAGCATGCTGGCGTTCTCCCCAGCTCAAGCAAGACTCTAGGT
ASI4169    CAAGCTGCAGCAGGCTCTTCTTCAACATCAGCATGCTGGCGTTCTCCCCAGCTCAAGCAAGACTCTAGGT

                   430       440       450       460       470       480       490
KACC42870  GTACTTCCTCTAATGGATCCGATGGCCGCGACAAGCATGCCTGAGGCTTGGGTTCGAGGTGCCATGCTAA
ASI4208    GTACTTCCTCTAATGGATCCGATGGCCGCGACAAGCATGCCTGAGGCTTGGGTTCGAGGTGCCATGCTAA
ASI4049    GTACTTCCTCTAATGGATCCGATGGCCGCGACAAGCATGCCTGAGGCTTGGGTTCGAGGTGCCATGCTAA
ASI4057    GTACTTCCTCTAATGGATCCGATGGCCGCGACAAGCATGCCTGAGGCTTGGGTTCGAGGTGCCATGCTAA
ASI4166    GTACTTCCTCTAATGGATCCGATGGCCGCGACAAGCATGCCTGAGGCTTGGGTTCGAGGTGCCATGCTAA
ASI4167    GTACTTCCTCTAATGGATCCGATGGCCGCGACAAGCATGCCTGAGGCTTGGGTTCGAGGTGCCATGCTAA
ASI4169    GTACTTCCTCTAATGGATCCGATGGCCGCGACAAGCATGCCTGAGGCTTGGGTTCGAGGTGCCATGCTAA

                   500       510       520       530       540       550       560
KACC42870  TCCGAATGAACTCGTTGATACGGGGGCATTCAGGAGTTCGATGGGAGCTTATTGAAAAGATCAATGACCT
ASI4208    TCCGAATGAACTCGTTGATACGGGGGCATTCAGGAGTTCGATGGGAGCTTATTGAAAAGATCAATGACCT
ASI4049    TCCGAATGAACTCGTTGATACGGGGGCATTCAGGAGTTCGATGGGAGCTTATTGAAAAGATCAATGACCT
ASI4057    TCCGAATGAACTCGTTGATACGGGGGCATTCAGGAGTTCGATGGGAGCTTATTGAAAAGATCAATGACCT
ASI4166    TCCGAATGAACTCGTTGATACGGGGGCATTCAGGAGTTCGATGGGAGCTTATTGAAAAGATCAATGACCT
ASI4167    TCCGAATGAACTCGTTGATACGGGGGCATTCAGGAGTTCGATGGGAGCTTATTGAAAAGATCAATGACCT
ASI4169    TCCGAATGAACTCGTTGATACGGGGGCATTCAGGAGTTCGATGGGAGCTTATTGAAAAGATCAATGACCT
```

```
                    570       580       590       600       610       620       630
KACC42870  GCTCCGAGCCAACATCACACCAGTAGTCCCGCTTCGCTCAAGCATCTCAGCCTCCGGAGATTTATCTCCT
ASI4208    GCTCCGAGCCAACATCACACCAGTAGTCCCGCTTCGCTCAAGCATCTCAGCCTCCGGAGATTTATCTCCT
ASI4049    GCTCCGAGCCAACATCACACCAGTAGTCCCGCTTCGCTCAAGCATCTCAGCCTCCGGAGATTTATCTCCT
ASI4057    GCTCCGAGCCAACATCACACCAGTAGTCCCGCTTCGCTCAAGCATCTCAGCCTCCGGAGATTTATCTCCT
ASI4166    GCTCCGAGCCAACATCACACCAGTAGTCCCGCTTCGCTCAAGCATCTCAGCCTCCGGAGATTTATCTCCT
ASI4167    GCTCCGAGCCAACATCACACCAGTAGTCCCGCTTCGCTCAAGCATCTCAGCCTCCGGAGATTTATCTCCT
ASI4169    GCTCCGAGCCAACATCACACCAGTAGTCCCGCTTCGCTCAAGCATCTCAGCCTCCGGAGATTTATCTCCT

                    640       650       660       670       680       690       700
KACC42870  CTCTCATATGTGGCTGGAACCTTGACCGCCAACCCATCGATTCGCGTCTTCGATGGACCTTCTGCCTTCG
ASI4208    CTCTCATATGTGGCTGGAACCTTGACCGCCAACCCATCGATTCGCGTCTTCGATGGACCTTCTGCCTTCG
ASI4049    CTCTCATATGTGGCTGGAACCTTGACCGCCAACCCATCGATTCGCGTCTTCGATGGACCTTCTGCCTTCG
ASI4057    CTCTCATATGTGGCTGGAACCTTGACCGCCAACCCATCGATTCGCGTCTTCGATGGACCTTCTGCCTTCG
ASI4166    CTCTCATATGTGGCTGGAACCTTGACCGCCAACCCATCGATTCGCGTCTTCGATGGACCTTCTGCCTTCG
ASI4167    CTCTCATATGTGGCTGGAACCTTGACCGCCAACCCATCGATTCGCGTCTTCGATGGACCTTCTGCCTTCG
ASI4169    CTCTCATATGTGGCTGGAACCTTGACCGCCAACCCATCGATTCGCGTCTTCGATGGACCTTCTGCCTTCG

                    710       720       730       740       750       760       770
KACC42870  GTGCGCGAAAAATGGTCTCATCTAGAGACGCTTTGGCCGCGCACAAGATCAAACCAGTCACTCTTGCGTC
ASI4208    GTGCGCGAAAAATGGTCTCATCTAGAGACGCTTTGGCTGCGCACAAGATCAAACCAGTCACTCTTGCGTC
ASI4049    GTGCGCGAAAAATGGTCTCATCTAGAGACGCTTTGGCCGCGCACAAGATCAAACCAGTCACTCTTGCGTC
ASI4057    GTGCGCGAAAAATGGTCTCATCTAGAGACGCTTTGGCCGCGCACAAGATCAAACCAGTCACTCTTGCGTC
ASI4166    GTGCGCGAAAAATGGTCTCATCTAGAGACGCTTTGGCTGCGCACAAGATCAAACCAGTCACTCTTGCGTC
ASI4167    GTGCGCGAAAAATGGTCTCATCTAGAGACGCTTTGGCTGCGCACAAGATCAAACCAGTCACTCTTGCGTC
ASI4169    GTGCGCGAAAAATGGTCTCATCTAGAGACGCTTTGGCTGCGCACAAGATCAAACCAGTCACTCTTGCGTC

                    780       790       800       810       820       830       840
KACC42870  CAAAGAGGGTTTAGGCATTTTGAACGGGACGGCATTTTCCGCGGCAGTCGCGTCACTTGCTCTGACTGAG
ASI4208    CAAAGAGGGTTTAGGCATTTTGAACGGGACGGCATTTTCCGCGGCAGTCGCGTCACTTGCTCTGACTGAG
ASI4049    CAAAGAGGGTTTAGGCATTTTGAACGGGACGGCATTTTCCGCGGCAGTCGCGTCACTTGCTCTGACTGAG
ASI4057    CAAAGAGGGTTTAGGCATTTTGAACGGGACGGCATTTTCCGCGGCAGTCGCGTCACTTGCTCTGACTGAG
ASI4166    CAAAGAGGGTTTAGGCATTTTGAACGGGACGGCATTTTCCGCGGCAGTCGCGTCACTTGCTCTGACTGAG
ASI4167    CAAAGAGGGTTTAGGCATTTTGAACGGGACGGCATTTTCCGCGGCAGTCGCGTCACTTGCTCTGACTGAG
ASI4169    CAAAGAGGGTTTAGGCATTTTGAACGGGACGGCATTTTCCGCGGCAGTCGCGTCACTTGCTCTGACTGAG

                    850       860       870       880       890       900       910
KACC42870  GCAACTCATCTTGCTCTTCTGGCTCAAGTCTGTACTGCTCTTGGCACGGAGGCTCTTTGTGGAACAACAG
ASI4208    GCAACTCATCTTGCTCTTCTGGCTCAAGTCTGTACTGCTCTTGGCACGGAGGCTCTTTGTGGAACAACAG
ASI4049    GCAACTCATCTTGCTCTTCTGGCTCAAGTCTGTACTGCTCTTGGCACGGAGGCTCTTTGTGGAACAACAG
ASI4057    GCAACTCATCTTGCTCTTCTGGCTCAAGTCTGTACTGCTCTTGGCACGGAGGCTCTTTGTGGAACAACAG
ASI4166    GCAACTCATCTTGCTCTTCTGGCTCAAGTCTGTACTGCTCTTGGCACGGAGGCTCTTTGTGGAACAACAG
ASI4167    GCAACTCATCTTGCTCTTCTGGCTCAAGTCTGTACTGCTCTTGGCACGGAGGCTCTTTGTGGAACAACAG
ASI4169    GCAACTCATCTTGCTCTTCTGGCTCAAGTCTGTACTGCTCTTGGCACGGAGGCTCTTTGTGGAACAACAG

                    920       930       940       950       960       970       980
KACC42870  GATCATATGCGCCTTTCATCCATGTCACTGCTCGGCCTCATCCTGGCCAGATCGAAGCCGCCAACAACAT
ASI4208    GATCATATGCGCCTTTCATCCATGTCACTGCTCGGCCTCATCCTGGCCAGATCGAAGCCGCCAACAACAT
ASI4049    GATCATATGCGCCTTTCATCCATGTCACTGCTCGGCCTCATCCTGGCCAGATCGAAGCCGCCAACAACAT
ASI4057    GATCATATGCGCCTTTCATCCATGTCACTGCTCGGCCTCATCCTGGCCAGATCGAAGCCGCCAACAACAT
ASI4166    GATCATATGCGCCTTTCATCCATGTCACTGCTCGGCCTCATCCTGGCCAGATCGAAGCCGCCAACAACAT
ASI4167    GATCATATGCGCCTTTCATCCATGTCACTGCTCGGCCTCATCCTGGCCAGATCGAAGCCGCCAACAACAT
ASI4169    GATCATATGCGCCTTTCATCCATGTCACTGCTCGGCCTCATCCTGGCCAGATCGAAGCCGCCAACAACAT

                    990      1000      1010      1020      1030      1040      1050
KACC42870  GTGGAATCTTCTGCAAGGCAGTAAACTTGCCTCCGGACACGAAGAGGAAGTGTCCATCAACCAAGACAAA
ASI4208    GTGGAATCTTCTGCAAGGCAGTAAACTTGCCTCCGGACACGAAGAGGAAGTGTCCATCAACCAAGACAAA
ASI4049    GTGGAATCTTCTGCAAGGCAGTAAACTTGCCTCCGGACACGAAGAGGAAGTGTCCATCAACCAAGACAAA
ASI4057    GTGGAATCTTCTGCAAGGCAGTAAACTTGCCTCCGGACACGAAGAGGAAGTGTCCATCAACCAAGACAAA
ASI4166    GTGGAATCTTCTGCAAGGCAGTAAACTTGCCTCCGGACACGAAGAGGAAGTGTCCATCAACCAAGACAAA
ASI4167    GTGGAATCTTCTGCAAGGCAGTAAACTTGCCTCCGGACACGAAGAGGAAGTGTCCATCAACCAAGACAAA
ASI4169    GTGGAATCTTCTGCAAGGCAGTAAACTTGCCTCCGGACACGAAGAGGAAGTGTCCATCAACCAAGACAAA

                   1060      1070      1080      1090      1100      1110      1120
KACC42870  TATGAGTTACGCCAGGACCGCTACCCATTGAGGACCTCGCCCCAATTCCTCGGCCCTCAAATAGAAGATA
ASI4208    TATGAGTTACGCCAGGACCGCTACCCATTGAGGACCTCGCCCCAATTCCTCGGCCCTCAAATAGAAGATA
ASI4049    TATGAGTTACGCCAGGACCGCTACCCATTGAGGACCTCGCCCCAATTCCTCGGTCCTCAAATAGAAGATA
ASI4057    TATGAGTTACGCCAGGACCGCTACCCATTGAGGACCTCGCCCCAATTCCTCGGTCCTCAAATAGAAGATA
ASI4166    TATGAGTTACGCCAGGACCGCTACCCATTGAGGACCTCGCCCCAATTCCTCGGCCCTCAAATAGAAGATA
ASI4167    TATGAGTTACGCCAGGACCGCTACCCATTGAGGACCTCGCCCCAATTCCTCGGCCCTCAAATAGAAGATA
ASI4169    TATGAGTTACGCCAGGACCGCTACCCATTGAGGACCTCGCCCCAATTCCTCGGCCCTCAAATAGAAGATA
```

```
                1130      1140      1150      1160      1170      1180      1190
KACC42870  TTCTCGCTGCACTAGCATCAGTCACTCAAGAATGTAACTCAACCACTGATAACCCGCTTGTTGATGGGAA
ASI4208    TTCTCGCTGCACTAGCATCAGTCACTCAAGAATGTAACTCAACCACTGATAACCCGCTTGTTGATGGGAA
ASI4049    TTCTCGCTGCACTAGCATCAGTCACTCAAGAATGTAACTCAACCACTGATAACCCGCTTGTTGATGGGAA
ASI4057    TTCTCGCTGCACTAGCATCAGTCACTCAAGAATGTAACTCAACCACTGATAACCCGCTTGTTGATGGGAA
ASI4166    TTCTCGCTGCACTAGCATCAGTCACTCAAGAATGTAACTCAACCACTGATAACCCGCTTGTTGATGGGAA
ASI4167    TTCTCGCTGCACTAGCATCAGTCACTCAAGAATGTAACTCAACCACTGATAACCCGCTTGTTGATGGGAA
ASI4169    TTCTCGCTGCACTAGCATCAGTCACTCAAGAATGTAACTCAACCACTGATAACCCGCTTGTTGATGGGAA

                1200      1210      1220      1230      1240      1250      1260
KACC42870  CACTGGTGAAGTTCACCACGGCGGCAACTTCCAAGCTATGGCTATCTCTAACGCCATGGAAAAGACACGT
ASI4208    CACTGGTGAAGTTCACCACGGCGGCAACTTCCAAGCTATGGCTATCTCTAACGCCATGGAAAAGACACGT
ASI4049    CACTGGTGAAGTTCACCACGGCGGCAACTTCCAAGCTATGGCTATCTCTAACGCCATGGAAAAGACACGT
ASI4057    CACTGGTGAAGTTCACCACGGCGGCAACTTCCAAGCTATGGCTATCTCTAACGCCATGGAAAAGACACGT
ASI4166    CACTGGTGAAGTTCACCACGGCGGCAACTTCCAAGCTATGGCTATCTCTAACGCCATGGAAAAGACACGT
ASI4167    CACTGGTGAAGTTCACCACGGCGGCAACTTCCAAGCTATGGCTATCTCTAACGCCATGGAAAAGACACGT
ASI4169    CACTGGTGAAGTTCACCACGGCGGCAACTTCCAAGCTATGGCTATCTCTAACGCCATGGAAAAGACACGT

                1270      1280      1290      1300      1310      1320      1330
KACC42870  CTTGCAGTGCATCACATCGGCAAGCTTATGTTTTCTCAAAGCACCGAACTGGTCAACCCCGCTATGAACC
ASI4208    CTTGCAGTGCATCACATCGGCAAGCTTATGTTTTCTCAAAGCACCGAACTGGTCAACCCCGCTATGAACC
ASI4049    CTTGCAGTGCATCACATCGGCAAGCTTATGTTTTCTCAAAGCACCGAACTGGTCAACCCCGCTATGAACC
ASI4057    CTTGCAGTGCATCACATCGGCAAGCTTATGTTTTCTCAAAGCACCGAACTGGTCAACCCCGCTATGAACC
ASI4166    CTTGCAGTGCATCACATCGGCAAGCTTATGTTTTCTCAAAGCACCGAACTGGTCAACCCCGCTATGAACC
ASI4167    CTTGCAGTGCATCACATCGGCAAGCTTATGTTTTCTCAAAGCACCGAACTGGTCAACCCCGCTATGAACC
ASI4169    CTTGCAGTGCATCACATCGGCAAGCTTATGTTTTCTCAAAGCACCGAACTGGTCAACCCCGCTATGAACC

                1340      1350      1360      1370      1380      1390      1400
KACC42870  ATGGCCTTCCGCCTTCGCTCGCTGCTTCTGATCCGTCTTTGAACTACCACGGCAAGGGTGTCGATATCGC
ASI4208    ATGGCCTTCCGCCTTCGCTCGCTGCTTCTGATCCGTCTTTGAACTACCACGGCAAGGGTGTCGATATCGC
ASI4049    ATGGCCTTCCGCCTTCGCTCGCTGCTTCTGATCCGTCTTTGAACTACCACGGCAAGGGTGTCGATATCGC
ASI4057    ATGGCCTTCCGCCTTCGCTCGCTGCTTCTGATCCGTCTTTGAACTACCACGGCAAGGGTGTCGATATCGC
ASI4166    ATGGCCTTCCGCCTTCGCTCGCTGCTTCTGATCCGTCTTTGAACTACCACGGCAAGGGTGTCGATATCGC
ASI4167    ATGGCCTTCCGCCTTCGCTCGCTGCTTCTGATCCGTCTTTGAACTACCACGGCAAGGGTGTCGATATCGC
ASI4169    ATGGCCTTCCGCCTTCGCTCGCTGCTTCTGATCCGTCTTTGAACTACCACGGCAAGGGTGTCGATATCGC

                1410      1420      1430      1440      1450      1460      1470
KACC42870  CACTGCAGCGTACGTCTCAGAGTTAGGCTACCTCGCAAACCCCGTCACCACACACATTCAATCCGCCGAG
ASI4208    CACTGCAGCGTACGTCTCAGAGTTAGGCTACCTCGCAAACCCCGTCACCACACACATTCAATCCGCCGAG
ASI4049    CACTGCAGCGTACGTCTCAGAGTTAGGCTACCTCGCAAACCCCGTCACCACACACATTCAATCCGCCGAG
ASI4057    CACTGCAGCGTACGTCTCAGAGTTAGGCTACCTCGCAAACCCCGTCACCACACACATTCAATCCGCCGAG
ASI4166    CACTGCAGCGTACGTCTCAGAGTTAGGCTACCTCGCAAACCCCGTCACCACACACATTCAATCCGCCGAG
ASI4167    CACTGCAGCGTACGTCTCAGAGTTAGGCTACCTCGCAAACCCCGTCACCACACACATTCAATCCGCCGAG
ASI4169    CACTGCAGCGTACGTCTCAGAGTTAGGCTACCTCGCAAACCCCGTCACCACACACATTCAATCCGCCGAG

                1480      1490      1500      1510      1520      1530      1540
KACC42870  ATGCACAACCAGGCTGTGAACTCGTTGGCTCTGATCTCTGCCCGAGCGACTGTGACTTCTCTGGATGTCC
ASI4208    ATGCACAACCAGGCTGTGAACTCGTTGGCTCTGATCTCTGCCCGAGCGACTGTGACTTCTCTGGATGTCC
ASI4049    ATGCACAACCAGGCTGTGAACTCGTTGGCTCTGATCTCTGCCCGAGCGACTGTGACTTCTCTGGATGTCC
ASI4057    ATGCACAACCAGGCTGTGAACTCGTTGGCTCTGATCTCTGCCCGAGCGACTGTGACTTCTCTGGATGTCC
ASI4166    ATGCACAACCAGGCTGTGAACTCGTTGGCTCTGATCTCTGCCCGAGCGACTGTGACTTCTCTGGATGTCC
ASI4167    ATGCACAACCAGGCTGTGAACTCGTTGGCTCTGATCTCTGCCCGAGCGACTGTGACTTCTCTGGATGTCC
ASI4169    ATGCACAACCAGGCTGTGAACTCGTTGGCTCTGATCTCTGCCCGAGCGACTGTGACTTCTCTGGATGTCC

                1550      1560      1570      1580      1590      1600      1610
KACC42870  TGACCATTCTTATGTCCTCGTACCTCTACCTCCTCTGCCAAGCTGTTGACCTTCGTGCCCTCCGACGCGA
ASI4208    TGACCATTCTTATGTCCTCGTACCTCTACCTCCTCTGCCAAGCTGTTGACCTTCGTGCCCTCCGACGCGA
ASI4049    TGACCATTCTTATGTCCTCGTACCTCTACCTCCTCTGCCAAGCTGTTGACCTTCGTGCCCTCCGACGCGA
ASI4057    TGACCATTCTTATGTCCTCGTACCTCTACCTCCTCTGCCAAGCTGTTGACCTTCGTGCCCTCCGACGCGA
ASI4166    TGACCATTCTTATGTCCTCGTACCTCTACCTCCTCTGCCAAGCTGTTGACCTTCGTGCCCTCCGACGCGA
ASI4167    TGACCATTCTTATGTCCTCGTACCTCTACCTCCTCTGCCAAGCTGTTGACCTTCGTGCCCTCCGACGCGA
ASI4169    TGACCATTCTTATGTCCTCGTACCTCTACCTCCTCTGCCAAGCTGTTGACCTTCGTGCCCTCCGACGCGA

                1620      1630      1640      1650      1660      1670      1680
KACC42870  CCTAGATGTCGGTGTCCGCGCCATTATCGCTGAAGAAGTGTCTAAGCTTTTCTCAAACAACCTCTCTTCT
ASI4208    CCTAGATGTCGGTGTCCGCGCCATTATCGCTGAAGAAGTGTCTAAGCTTTTCTCAAACAACCTCTCTTCT
ASI4049    CCTAGATGTCGGTGTCCGCGCCATTATCGCTGAAGAAGTGTCTAAGCTTTTCTCAAACAACCTCTCTTCT
ASI4057    CCTAGATGTCGGTGTCCGCGCCATTATCGCTGAAGAAGTGTCTAAGCTTTTCTCAAACAACCTCTCTTCT
ASI4166    CCTAGATGTCGGTGTCCGCGCCATTATCGCTGAAGAAGTGTCTAAGCTTTTCTCAAACAACCTCTCTTCT
ASI4167    CCTAGATGTCGGTGTCCGCGCCATTATCGCTGAAGAAGTGTCTAAGCTTTTCTCAAACAACCTCTCTTCT
ASI4169    CCTAGATGTCGGTGTCCGCGCCATTATCGCTGAAGAAGTGTCTAAGCTTTTCTCAAACAACCTCTCTTCT
```

```
                   1690      1700      1710      1720      1730      1740      1750
           ....|....|....|....|....|....|....|....|....|....|....|....|....|....|
KACC42870  GAGGAAATGGACCTGCTACACTCATCCCTATATTCAAAATACCAACATACTATGGATAAGACGACCACAA
ASI4208    GAGGAAATGGACCTGCTACACTCATCCCTATATTCAAAATACCAACATACTATGGATAAGACGACCACAA
ASI4049    GAGGAAATGGACCTGCTACACTCATCCCTATATTCAAAATACCAGCATACTATGGATAAGACGACCACAA
ASI4057    GAGGAAATGGACCTGCTACACTCATCCCTCTATTCAAAATACCAGCATACTATGGATAAGACGACCACAA
ASI4166    GAGGAAATGGACCTGCTACACTCATCCCTATATTCAAAATACCAACATACTATGGATAAGACGACCACAA
ASI4167    GAGGAAATGGACCTGCTACACTCATCCCTATATTCAAAATACCAACATACTATGGATAAGACGACCACAA
ASI4169    GAGGAAATGGACCTGCTACACTCATCCCTATATTCAAAATACCAACATACTATGGATAAGACGACCACAA

                   1760      1770      1780      1790      1800      1810      1820
           ....|....|....|....|....|....|....|....|....|....|....|....|....|....|
KACC42870  TGGACGCCGTGGACCAGATGAAGGAGGTGACCGCATCTTTCGCGCCGATGCTCGTGGAGGTGTTCACCTC
ASI4208    TGGACGCCGTGGACCAGATGAAGGAGGTGACCGCATCTTTCGCGCCGATGCTCGTGGAGGTGTTCACCTC
ASI4049    TGGACGCCGTGGACCAGATGAAGGAGGTGACCGCATCTTTCGCGCCGATGCTCGTGGAGGTGTTCACCTC
ASI4057    TGGACGCCGTGGACCAGATGAAGGAGGTGACCGCATCTTTCGCGCCGATGCTCGTGGAGGTGTTCACCTC
ASI4166    TGGACGCCGTGGACCAGATGAAGGAGGTGACCGCATCTTTCGCGCCGATGCTCGTGGAGGTGTTCACCTC
ASI4167    TGGACGCCGTGGACCAGATGAAGGAGGTGACCGCATCTTTCGCGCCGATGCTCGTGGAGGTGTTCACCTC
ASI4169    TGGACGCCGTGGACCAGATGAAGGAGGTGACCGCATCTTTCGCGCCGATGCTCGTGGAGGTGTTCACCTC

                   1830      1840      1850      1860      1870      1880      1890
           ....|....|....|....|....|....|....|....|....|....|....|....|....|....|
KACC42870  AACTCGAGTCATGCCTGACGCCCTCAGCGCCATCCCTCGCTTTAGATCTAATATCTCTTCGCGCGCTACG
ASI4208    AACTCGAGTCATGCCTGACGCCCTCAGCGCCATCCCTCGCTTTAGATCTAATATCTCTTCGCGCGCTACG
ASI4049    AACTCGAGTCATGCCTGACGCCCTCAGCGCCATCCCTCGCTTTAGATCTAATATCTCTTCGCGCGCTACG
ASI4057    AACTCGAGTCATGCCTGACGCCCTCAGCGCCATCCCTCGCTTTAGATCTAATATCTCTTCGCGCGCTACG
ASI4166    AACTCGAGTCATGCCTGACGCCCTCAGCGCCATCCCTCGCTTTAGATCTAATATCTCTTCGCGCGCTACG
ASI4167    AACTCGAGTCATGCCTGACGCCCTCAGCGCCATCCCTCGCTTTAGATCTAATATCTCTTCGCGCGCTACG
ASI4169    AACTCGAGTCATGCCTGACGCCCTCAGCGCCATCCCTCGCTTTAGATCTAATATCTCTTCGCGCGCTACG

                   1900      1910      1920      1930      1940      1950      1960
           ....|....|....|....|....|....|....|....|....|....|....|....|....|....|
KACC42870  CAGCTGTTTGATAGACTACGCGCAAGCTACCTTTCGGGCGAGCGTGGTGCCACTCCTGCCAGCTCCCTTT
ASI4208    CAGCTGTTTGATAGACTACGCGCAAGCTACCTTTCGGGCGAGCGTGGTGCCACTCCTGCCAGCTCCCTTT
ASI4049    CAGCTGTTTGATAGACTACGCGCAAGCTACCTTTCGGGCGAGCGTGGTGCCACTCCTGCCAGCTCCCTTT
ASI4057    CAGCTGTTTGATAGACTACGCGCAAGCTACCTTTCGGGCGAGCGTGGTGCCACTCCTGCCAGCTCCCTTT
ASI4166    CAGCTGTTTGATAGACTACGCGCAAGCTACCTTTCGGGCGAGCGTGGTGCCACTCCTGCCAGCTCCCTTT
ASI4167    CAGCTGTTTGATAGACTACGCGCAAGCTACCTTTCGGGCGAGCGTGGTGCCACTCCTGCCAGCTCCCTTT
ASI4169    CAGCTGTTTGATAGACTACGCGCAAGCTACCTTTCGGGCGAGCGTGGTGCCACTCCTGCCAGCTCCCTTT

                   1970      1980      1990      2000      2010      2020      2030
           ....|....|....|....|....|....|....|....|....|....|....|....|....|....|
KACC42870  TAGGCCGGACACGTTCGGTGTACGAATTCATCCGTGTCTCTTTGGGAATTCGCATGCATGGCTCGGAGAA
ASI4208    TAGGCCGGACACGTTCGGTGTACGAATTCATCCGTGTCTCTTTGGGAATTCGCATGCATGGCTCGGAGAA
ASI4049    TAGGCCGGACACGTTCGGTGTACGAATTCATCCGTGTCTCTTTGGGAATTCGCATGCATGGCTCGGAGAA
ASI4057    TAGGCCGGACACGTTCGGTGTACGAATTCATCCGTGTCTCTTTGGGAATTCGCATGCATGGCTCGGAGAA
ASI4166    TAGGCCGGACACGTTCGGTGTACGAATTCATCCGTGTCTCTTTGGGAATTCGCATGCATGGCTCGGAGAA
ASI4167    TAGGCCGGACACGTTCGGTGTACGAATTCATCCGTGTCTCTTTGGGAATTCGCATGCATGGCTCGGAGAA
ASI4169    TAGGCCGGACACGTTCGGTGTACGAATTCATCCGTGTCTCTTTGGGAATTCGCATGCATGGCTCGGAGAA

                   2040      2050      2060      2070      2080      2090      2100
           ....|....|....|....|....|....|....|....|....|....|....|....|....|....|
KACC42870  CTATAGCGCCTTTGCCAATGGGCTGGGCGTCGATGACCCTACTATTGGTCAGAACATCTCGTCGATCTAT
ASI4208    CTATAGCGCCTTTGCCAATGGGCTGGGCGTCGATGACCCTACTATTGGTCAGAACATCTCGTCGATCTAT
ASI4049    CTATAGCGCCTTTGCCAATGGGCTGGGCGTCGATGACCCTACTATTGGTCAGAACATCTCGTCGATCTAT
ASI4057    CTATAGCGCCTTTGCCAATGGGCTGGGCGTCGATGACCCTACTATTGGTCAGAACATCTCGTCGATCTAT
ASI4166    CTATAGCGCCTTTGCCAATGGGCTGGGCGTCGATGACCCTACTATTGGTCAGAACATCTCGTCGATCTAT
ASI4167    CTATAGCGCCTTTGCCAATGGGCTGGGCGTCGATGACCCTACTATTGGTCAGAACATCTCGTCGATCTAT
ASI4169    CTATAGCGCCTTTGCCAATGGGCTGGGCGTCGATGACCCTACTATTGGTCAGAACATCTCGTCGATCTAT

                   2110      2120      2130      2140      2150      2160      2170
           ....|....|....|....|....|....|....|....|....|....|....|....|....|....|
KACC42870  GAGGCCATCCGGGACGGCAAATTCCACGACGTCGTAGCAGACCTCTTTGAGGCTCTGCCTCGCAGCAAGC
ASI4208    GAGGCCATCCGGGACGGCAAATTCCACGACGTCGTAGCAGACCTCTTTGAGGCTCTGCCTCGCAGCAAGC
ASI4049    GAGGCCATCCGGGACGGCAAATTCCACGACGTCGTAGCAGACCTCTTTGAGGCTCTGCCTCGCAGCAAGC
ASI4057    GAGGCCATCCGGGACGGCAAATTCCACGACGTCGTAGCAGACCTCTTTGAGGCTCTGCCTCGCAGCAAGC
ASI4166    GAGGCCATCCGGGACGGCAAATTCCACGACGTCGTAGCAGACCTCTTTGAGGCTCTGCCTCGCAGCAAGC
ASI4167    GAGGCCATCCGGGACGGCAAATTCCACGACGTCGTAGCAGACCTCTTTGAGGCTCTGCCTCGCAGCAAGC
ASI4169    GAGGCCATCCGGGACGGCAAATTCCACGACGTCGTAGCAGACCTCTTTGAGGCTCTGCCTCGCAGCAAGC

           ....|
KACC42870  TCTAG
ASI4208    TCTAG
ASI4049    TCTAG
ASI4057    TCTAG
ASI4166    TCTAG
ASI4167    TCTAG
ASI4169    TCTAG
```

(a)

```
                   10        20        30        40        50        60        70
           ....|....|....|....|....|....|....|....|....|....|....|....|....|....|
KACC42870  MPSELFDLDNARAARDTFLDARRTATLLHKFLDSHRELKSYKNGRTINVDGHTLSLAAVTAAARYNANVE
ASI4208    MPSELFDLDNARAARDTFLDARRTATLLHKFLDSHRELKSYKNGRTINVDGHTLSLAAVTAAARYNANVE
ASI4049    MPSELFDLDNARAARDTFLDARRTATLLHKFLDSHRELKSYKNGRTINVDGHTLSLAAVTAAARYNANVE
ASI4057    MPSELFDLDNARAARDTFLDARRTATLLHKFLDSHRELKSYKNGRTINVDGHTLSLAAVTAAARYNANVE
ASI4166    MPSELFDLDNARAARDTFLDARRTATLLHKFLDSHRELKSYKNGRTINVDGHTLSLAAVTAAARYNANVE
ASI4167    MPSELFDLDNARAARDTFLDARRTATLLHKFLDSHRELKSYKNGRTINVDGHTLSLAAVTAAARYNANVE
ASI4169    MPSELFDLDNARAARDTFLDARRTATLLHKFLDSHRELKSYKNGRTINVDGHTLSLAAVTAAARYNANVE

                   80        90       100       110       120       130       140
           ....|....|....|....|....|....|....|....|....|....|....|....|....|....|
KACC42870  LSQSAQVKEGVEKSRAVIAEKVEQGTSVYGVSTGFGGSADTRTDQPLKLQQALLQHQHAGVLPSSSKTLG
ASI4208    LSQSAQVKEGVEKSRAVIAEKVEQGTSVYGVSTGFGGSADTRTDQPLKLQQALLQHQHAGVLPSSSKTLG
ASI4049    LSQSAQVKEGVEKSRAVIAEKVEQGTSVYGVSTGFGGSADTRTDQPLKLQQALLQHQHAGVLPSSSKTLG
ASI4057    LSQSAQVKEGVEKSRAVIAEKVEQGTSVYGVSTGFGGSADTRTDQPLKLQQALLQHQHAGVLPSSSKTLG
ASI4166    LSQSAQVKEGVEKSRAVIAEKVEQGTSVYGVSTGFGGSADT--DQPLKLQQALLQHQHAGVLPSSSKTLG
ASI4167    LSQSAQVKEGVEKSRAVIAEKVEQGTSVYGVSTGFGGSADT--DQPLKLQQALLQHQHAGVLPSSSKTLG
ASI4169    LSQSAQVKEGVEKSRAVIAEKVEQGTSVYGVSTGFGGSADT--DQPLKLQQALLQHQHAGVLPSSSKTLG

                  150       160       170       180       190       200       210
           ....|....|....|....|....|....|....|....|....|....|....|....|....|....|
KACC42870  VLPLMDPMAATSMPEAWVRGAMLIRMNSLIRGHSGVRWELIEKINDLLRANITPVVPLRSSISASGDLSP
ASI4208    VLPLMDPMAATSMPEAWVRGAMLIRMNSLIRGHSGVRWELIEKINDLLRANITPVVPLRSSISASGDLSP
ASI4049    VLPLMDPMAATSMPEAWVRGAMLIRMNSLIRGHSGVRWELIEKINDLLRANITPVVPLRSSISASGDLSP
ASI4057    VLPLMDPMAATSMPEAWVRGAMLIRMNSLIRGHSGVRWELIEKINDLLRANITPVVPLRSSISASGDLSP
ASI4166    VLPLMDPMAATSMPEAWVRGAMLIRMNSLIRGHSGVRWELIEKINDLLRANITPVVPLRSSISASGDLSP
ASI4167    VLPLMDPMAATSMPEAWVRGAMLIRMNSLIRGHSGVRWELIEKINDLLRANITPVVPLRSSISASGDLSP
ASI4169    VLPLMDPMAATSMPEAWVRGAMLIRMNSLIRGHSGVRWELIEKINDLLRANITPVVPLRSSISASGDLSP

                  220       230       240       250       260       270       280
           ....|....|....|....|....|....|....|....|....|....|....|....|....|....|
KACC42870  LSYVAGTLTANPSIRVFDGPSAFGARKMVSSRDALAAHKIKPVTLASKEGLGILNGTAFSAAVASLALTE
ASI4208    LSYVAGTLTANPSIRVFDGPSAFGARKMVSSRDALAAHKIKPVTLASKEGLGILNGTAFSAAVASLALTE
ASI4049    LSYVAGTLTANPSIRVFDGPSAFGARKMVSSRDALAAHKIKPVTLASKEGLGILNGTAFSAAVASLALTE
ASI4057    LSYVAGTLTANPSIRVFDGPSAFGARKMVSSRDALAAHKIKPVTLASKEGLGILNGTAFSAAVASLALTE
ASI4166    LSYVAGTLTANPSIRVFDGPSAFGARKMVSSRDALAAHKIKPVTLASKEGLGILNGTAFSAAVASLALTE
ASI4167    LSYVAGTLTANPSIRVFDGPSAFGARKMVSSRDALAAHKIKPVTLASKEGLGILNGTAFSAAVASLALTE
ASI4169    LSYVAGTLTANPSIRVFDGPSAFGARKMVSSRDALAAHKIKPVTLASKEGLGILNGTAFSAAVASLALTE

                  290       300       310       320       330       340       350
           ....|....|....|....|....|....|....|....|....|....|....|....|....|....|
KACC42870  ATHLALLAQVCTALGTEALCGTTGSYAPFIHVTARPHPGQIEAANNMWNLLQGSKLASGHEEEVSINQDK
ASI4208    ATHLALLAQVCTALGTEALCGTTGSYAPFIHVTARPHPGQIEAANNMWNLLQGSKLASGHEEEVSINQDK
ASI4049    ATHLALLAQVCTALGTEALCGTTGSYAPFIHVTARPHPGQIEAANNMWNLLQGSKLASGHEEEVSINQDK
ASI4057    ATHLALLAQVCTALGTEALCGTTGSYAPFIHVTARPHPGQIEAANNMWNLLQGSKLASGHEEEVSINQDK
ASI4166    ATHLALLAQVCTALGTEALCGTTGSYAPFIHVTARPHPGQIEAANNMWNLLQGSKLASGHEEEVSINQDK
ASI4167    ATHLALLAQVCTALGTEALCGTTGSYAPFIHVTARPHPGQIEAANNMWNLLQGSKLASGHEEEVSINQDK
ASI4169    ATHLALLAQVCTALGTEALCGTTGSYAPFIHVTARPHPGQIEAANNMWNLLQGSKLASGHEEEVSINQDK

                  360       370       380       390       400       410       420
           ....|....|....|....|....|....|....|....|....|....|....|....|....|....|
KACC42870  YELRQDRYPLRTSPQFLGPQIEDILAALASVTQECNSTTDNPLVDGNTGEVHHGGNFQAMAISNAMEKTR
ASI4208    YELRQDRYPLRTSPQFLGPQIEDILAALASVTQECNSTTDNPLVDGNTGEVHHGGNFQAMAISNAMEKTR
ASI4049    YELRQDRYPLRTSPQFLGPQIEDILAALASVTQECNSTTDNPLVDGNTGEVHHGGNFQAMAISNAMEKTR
ASI4057    YELRQDRYPLRTSPQFLGPQIEDILAALASVTQECNSTTDNPLVDGNTGEVHHGGNFQAMAISNAMEKTR
ASI4166    YELRQDRYPLRTSPQFLGPQIEDILAALASVTQECNSTTDNPLVDGNTGEVHHGGNFQAMAISNAMEKTR
ASI4167    YELRQDRYPLRTSPQFLGPQIEDILAALASVTQECNSTTDNPLVDGNTGEVHHGGNFQAMAISNAMEKTR
ASI4169    YELRQDRYPLRTSPQFLGPQIEDILAALASVTQECNSTTDNPLVDGNTGEVHHGGNFQAMAISNAMEKTR

                  430       440       450       460       470       480       490
           ....|....|....|....|....|....|....|....|....|....|....|....|....|....|
KACC42870  LAVHHIGKLMFSQSTELVNPAMNHGLPPSLAASDPSLNYHGKGVDIATAAYVSELGYLANPVTTHIQSAE
ASI4208    LAVHHIGKLMFSQSTELVNPAMNHGLPPSLAASDPSLNYHGKGVDIATAAYVSELGYLANPVTTHIQSAE
ASI4049    LAVHHIGKLMFSQSTELVNPAMNHGLPPSLAASDPSLNYHGKGVDIATAAYVSELGYLANPVTTHIQSAE
ASI4057    LAVHHIGKLMFSQSTELVNPAMNHGLPPSLAASDPSLNYHGKGVDIATAAYVSELGYLANPVTTHIQSAE
ASI4166    LAVHHIGKLMFSQSTELVNPAMNHGLPPSLAASDPSLNYHGKGVDIATAAYVSELGYLANPVTTHIQSAE
ASI4167    LAVHHIGKLMFSQSTELVNPAMNHGLPPSLAASDPSLNYHGKGVDIATAAYVSELGYLANPVTTHIQSAE
ASI4169    LAVHHIGKLMFSQSTELVNPAMNHGLPPSLAASDPSLNYHGKGVDIATAAYVSELGYLANPVTTHIQSAE

                  500       510       520       530       540       550       560
           ....|....|....|....|....|....|....|....|....|....|....|....|....|....|
KACC42870  MHNQAVNSLALISARATVTSLDVLTILMSSYLYLLCQAVDLRALRRDLDVGVRAIIAEEVSKLFSNNLSS
ASI4208    MHNQAVNSLALISARATVTSLDVLTILMSSYLYLLCQAVDLRALRRDLDVGVRAIIAEEVSKLFSNNLSS
ASI4049    MHNQAVNSLALISARATVTSLDVLTILMSSYLYLLCQAVDLRALRRDLDVGVRAIIAEEVSKLFSNNLSS
ASI4057    MHNQAVNSLALISARATVTSLDVLTILMSSYLYLLCQAVDLRALRRDLDVGVRAIIAEEVSKLFSNNLSS
ASI4166    MHNQAVNSLALISARATVTSLDVLTILMSSYLYLLCQAVDLRALRRDLDVGVRAIIAEEVSKLFSNNLSS
ASI4167    MHNQAVNSLALISARATVTSLDVLTILMSSYLYLLCQAVDLRALRRDLDVGVRAIIAEEVSKLFSNNLSS
ASI4169    MHNQAVNSLALISARATVTSLDVLTILMSSYLYLLCQAVDLRALRRDLDVGVRAIIAEEVSKLFSNNLSS
```

```
                 570       580       590       600       610       620       630
KACC42870   EEMDLLHSSLYSKYQHTMDKTTTMDAVDQMKEVTASFAPMLVEVFTSTRVMPDALSAIPRFRSNISSRAT
ASI4208     EEMDLLHSSLYSKYQHTMDKTTTMDAVDQMKEVTASFAPMLVEVFTSTRVMPDALSAIPRFRSNISSRAT
ASI4049     EEMDLLHSSLYSKYQHTMDKTTTMDAVDQMKEVTASFAPMLVEVFTSTRVMPDALSAIPRFRSNISSRAT
ASI4057     EEMDLLHSSLYSKYQHTMDKTTTMDAVDQMKEVTASFAPMLVEVFTSTRVMPDALSAIPRFRSNISSRAT
ASI4166     EEMDLLHSSLYSKYQHTMDKTTTMDAVDQMKEVTASFAPMLVEVFTSTRVMPDALSAIPRFRSNISSRAT
ASI4167     EEMDLLHSSLYSKYQHTMDKTTTMDAVDQMKEVTASFAPMLVEVFTSTRVMPDALSAIPRFRSNISSRAT
ASI4169     EEMDLLHSSLYSKYQHTMDKTTTMDAVDQMKEVTASFAPMLVEVFTSTRVMPDALSAIPRFRSNISSRAT

                 640       650       660       670       680       690       700
KACC42870   QLFDRLRASYLSGERGATPASSLLGRTRSVYEFIRVSLGIRMHGSENYSAFANGLGVDDPTIGQNISSIY
ASI4208     QLFDRLRASYLSGERGATPASSLLGRTRSVYEFIRVSLGIRMHGSENYSAFANGLGVDDPTIGQNISSIY
ASI4049     QLFDRLRASYLSGERGATPASSLLGRTRSVYEFIRVSLGIRMHGSENYSAFANGLGVDDPTIGQNISSIY
ASI4057     QLFDRLRASYLSGERGATPASSLLGRTRSVYEFIRVSLGIRMHGSENYSAFANGLGVDDPTIGQNISSIY
ASI4166     QLFDRLRASYLSGERGATPASSLLGRTRSVYEFIRVSLGIRMHGSENYSAFANGLGVDDPTIGQNISSIY
ASI4167     QLFDRLRASYLSGERGATPASSLLGRTRSVYEFIRVSLGIRMHGSENYSAFANGLGVDDPTIGQNISSIY
ASI4169     QLFDRLRASYLSGERGATPASSLLGRTRSVYEFIRVSLGIRMHGSENYSAFANGLGVDDPTIGQNISSIY

                 710       720
KACC42870   EAIRDGKFHDVVADLFEALPRSKLX
ASI4208     EAIRDGKFHDVVADLFEALPRSKLX
ASI4049     EAIRDGKFHDVVADLFEALPRSKLX
ASI4057     EAIRDGKFHDVVADLFEALPRSKLX
ASI4166     EAIRDGKFHDVVADLFEALPRSKLX
ASI4167     EAIRDGKFHDVVADLFEALPRSKLX
ASI4169     EAIRDGKFHDVVADLFEALPRSKLX
```

(b)

Figure S1. Alignments of phenylalanine ammonia-lyase 1 (*Fvpal1*) genes of *Flammulina velutipes* strains. (**a**) cDNA sequences; (**b**) amino acid sequences. *F. velutipes* KACC42870 (non-white strain), *F. velutipes* ASI4208 (non-white strain), *F. velutipes* ASI4049 (non-white strain), *F. velutipes* ASI4057 (non-white strain), *F. velutipes* ASI4166 (white strain), *F. velutipes* ASI4167 (white strain), *F. velutipes* ASI4169 (white strain).
